# Whole-Genome Resequencing and Pan-Transcriptome Reconstruction Highlight the Impact of Genomic Structural Variation on Secondary Metabolite Gene Clusters in the Grapevine Esca Pathogen *Phaeoacremonium minimum*

**DOI:** 10.3389/fmicb.2018.01784

**Published:** 2018-08-13

**Authors:** Mélanie Massonnet, Abraham Morales-Cruz, Andrea Minio, Rosa Figueroa-Balderas, Daniel P. Lawrence, Renaud Travadon, Philippe E. Rolshausen, Kendra Baumgartner, Dario Cantu

**Affiliations:** ^1^Department of Viticulture and Enology, University of California, Davis, Davis, CA, United States; ^2^Department of Plant Pathology, University of California, Davis, Davis, CA, United States; ^3^Department of Botany and Plant Sciences, University of California, Riverside, Riverside, CA, United States; ^4^Crops Pathology and Genetics Research Unit, Agricultural Research Service, United States Department of Agriculture, Davis, CA, United States

**Keywords:** Esca, pathogenomics, comparative genomics, pan-transcriptome, structural variation, intraspecific genetic diversity, secondary metabolism

## Abstract

The Ascomycete fungus *Phaeoacremonium minimum* is one of the primary causal agents of Esca, a widespread and damaging grapevine trunk disease. Variation in virulence among *Pm. minimum* isolates has been reported, but the underlying genetic basis of the phenotypic variability remains unknown. The goal of this study was to characterize intraspecific genetic diversity and explore its potential impact on virulence functions associated with secondary metabolism, cellular transport, and cell wall decomposition. We generated a chromosome-scale genome assembly, using single molecule real-time sequencing, and resequenced the genomes and transcriptomes of multiple isolates to identify sequence and structural polymorphisms. Numerous insertion and deletion events were found for a total of about 1 Mbp in each isolate. Structural variation in this extremely gene dense genome frequently caused presence/absence polymorphisms of multiple adjacent genes, mostly belonging to biosynthetic clusters associated with secondary metabolism. Because of the observed intraspecific diversity in gene content due to structural variation we concluded that a transcriptome reference developed from a single isolate is insufficient to represent the virulence factor repertoire of the species. We therefore compiled a pan-transcriptome reference of *Pm. minimum* comprising a non-redundant set of 15,245 protein-coding sequences. Using naturally infected field samples expressing Esca symptoms, we demonstrated that mapping of meta-transcriptomics data on a multi-species reference that included the *Pm. minimum* pan-transcriptome allows the profiling of an expanded set of virulence factors, including variable genes associated with secondary metabolism and cellular transport.

## Introduction

**FIGURE 1:**
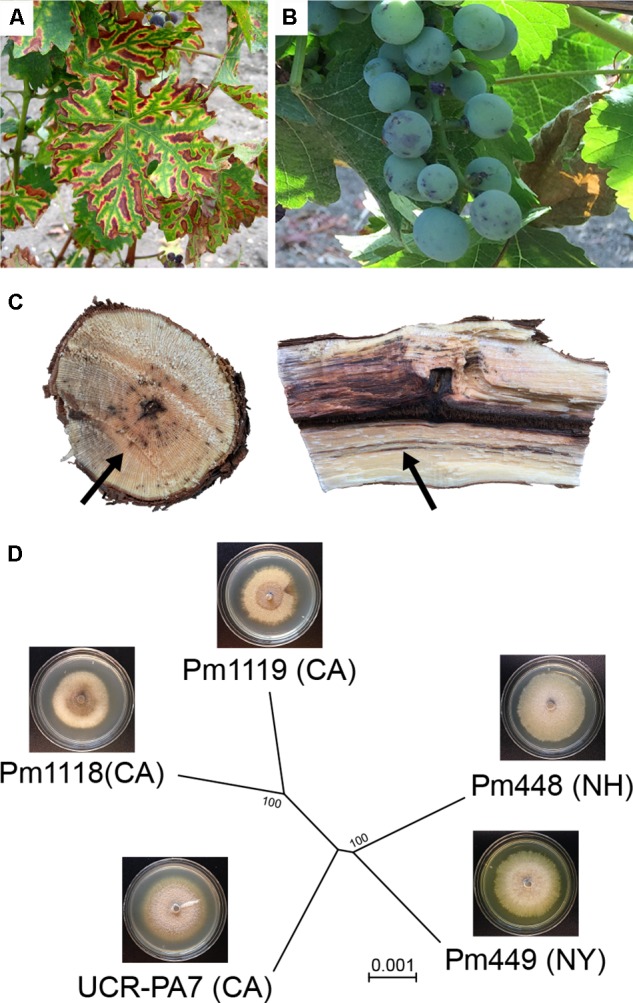
Esca symptoms in grapevine and phylogenetic relation of the five *Phaeoacremonium minimum* isolates used in this study. **(A)** Typical foliar symptoms of Esca in a red grape cultivar, **(B)** berry spotting (*measles*) and **(C)** black streaking (*arrows*) caused by wood colonization of Esca pathogens. **(D)** Dendrogram illustrating that Pm1119 and Pm448 clustered with Pm1118 and Pm449, respectively, reflecting their geographic origins. The dendrogram was constructed with MEGA7 (Kumar et al., 2016) using the Neighbor-Joining method (Saitou and Nei, 1987) and was based on a total of 22,242,282 positions. Bootstrap confidence values (100 replicates) are shown next to the branches (Felsenstein, 1985). The evolutionary distances were computed using the Maximum Composite Likelihood method (Tamura et al., 2004) and are in the units of the number of base substitutions per site. The tree was visualized with FigTree v.1.4.3 (http://tree.bio.ed.ac.uk/software/figtree). Insets show images of *in vitro* cultures of the five isolates.

Grapevine trunk diseases (Esca, and Botryosphaeria-, Eutypa-, and Phomopsis-diebacks) are a significant threat to viticulture worldwide (Gramaje et al., 2018). They are caused by fungal pathogens that colonize the woody organs of grapevines and, by progressively damaging the vascular tissue, reduce yield and shorten the life span of the infected plant (Kaplan et al., 2016). Esca is one of the most destructive trunk diseases (Mostert et al., 2006; Larignon et al., 2009). Its symptoms include an interveinal discoloration and scorching of leaves (“tiger-stripe," **Figure 1A**), delayed bud break and dieback of shoot tips, formation of black spots on berries (“measles," **Figure 1B**), black lines or spots in the wood (**Figure 1C**), and, in severe cases, sudden wilting and collapse of the whole plant, also known as vine “apoplexy" (Mugnai et al., 1999; Surico et al., 2008; Gubler et al., 2015).

Esca is caused by a complex of fungal species, among which are the Ascomycetes *Phaeoacremonium minimum* and *Phaeomoniella chlamydospora* and Basidiomycetes, such as *Fomitiporia mediterranea* (Fischer, 2006; Surico et al., 2008; Cloete et al., 2014). Esca symptoms are thought to be due to the combined activities of phytotoxic metabolites and cell wall-degrading proteins secreted by the pathogens (Mugnai et al., 1999; Andolfi et al., 2011). *Phaeoacremonium minimum* is known to produce several phytotoxic secondary metabolites, including α-glucans and naphthalenone pentaketides, such as scytalone and isosclerone (Bruno and Sparapano, 2006a,b). In addition to phytotoxins, *Pm. minimum* secretes extracellular enzymes that degrade cell wall polysaccharides, such as xylanase, exo- and endo-β-1,4-glucanase and β-glucosidase (Valtaud et al., 2009). Previous analyses of a draft genome assembly of *Pm. minimum* provided a glimpse of the large number and broad diversity of genes involved in secondary metabolism and cell wall degradation (Blanco-Ulate et al., 2013; Morales-Cruz et al., 2015). Gene families of these putative virulence factors have undergone distinctive patterns of expansion and contraction in *Pm. minimum* and another Esca pathogen *Ph. chlamydospora*, relative to the genomes of other trunk pathogens, which may explain the differences between Esca symptoms and those of the dieback-type trunk diseases (Morales-Cruz et al., 2015).

Significant variability in virulence is reported among *Pm. minimum* isolates (Billones-Baaijens et al., 2013; Gramaje et al., 2013; Pitt et al., 2013; Pathrose et al., 2014). This phenotypic variability may reflect the considerable genetic variation at the population level in *Pm. minimum*, which has been described both at the vineyard scale (Peìros et al., 2000; Tegli et al., 2000; Borie et al., 2002) and between distant grape regions (Cottral et al., 2001; Martiìn and Martiìn, 2010; Gramaje et al., 2013). Genetic variation in *Pm. minimum* is likely due to its heterothallic reproductive system (Rooney-Latham et al., 2005). Indeed, sexual fruiting structures (perithecia) are produced in nature and sexual spores (ascospores) may be important for long-distance dispersal. *Pm. minimum* can also reproduce asexually via production of asexual spores (conidia), which may increase mutation rates, and thus genetic variation, as seen in conidiating lineages of the heterothallic Ascomycete fungus *Neurospora* (Nygren et al., 2011).

The impact of this genetic variation on *Pm. minimum* virulence functions remains unknown. In fungal pathogens, single nucleotide polymorphisms (SNPs) and chromosomal structural rearrangements have been shown to underlie gains in pathogenicity, virulence, or adaptation to new environments (Möller and Stukenbrock, 2017). SNPs, for example, may contribute to the generation and maintenance of allelic diversity, which characterizes patterns of host-pathogen co-evolution (Karasov et al., 2014; Genissel et al., 2017). Structural variations, such as insertions, deletions, and inversions, contribute to phenotypic variation and adaptation by modification of gene dosage, gene expression, or disruption of genes that span boundaries of structural rearrangements (Qutob et al., 2009; Chuma et al., 2011; Chow et al., 2012; Jones et al., 2014). For example, subtelomeric tandem duplications yielded a dramatic copy number increase of an arsenite efflux transporter conferring arsenite tolerance in *Cryptococcus neoformans* (Chow et al., 2012). Similarly, *Erysiphe necator* populations evolved increased fungicide tolerance to triazole fungicides as a result of multiple duplications of the *Cyp51* gene (Jones et al., 2014). Gene duplication and interchromosomal DNA exchange could also lead to formation of novel gene clusters, which may provide an adaptive advantage, as in the case of the *DAL* cluster in yeast (Wong and Wolfe, 2005).

In this study, we investigated the impact that structural variants have on putative virulence functions in *Pm. minimum*. We assembled a chromosome-scale and complete genome of a *Pm. minimum* isolate and resequenced at high-coverage the whole genomes of four additional isolates. We also sequenced the RNA of all isolates grown under different culture conditions, to generate a comprehensive representation of their transcriptomes and expression dynamics. Comparative genome and transcriptome analyses enabled identification of extensive structural variation. Deletions and insertions, in this remarkably gene-dense genome, resulted in hundreds of protein-coding genes that were not shared among isolates. These presence/absence polymorphisms often involved blocks of multiple adjacent virulence factors. Because the variable fraction of the *P. minimum* genome was enriched in clusters associated with secondary metabolism, we hypothesized that acquisition or loss of secondary metabolism functions has an adaptive effect on fitness. Finally, we incorporated all core and variable transcripts into a pan-transcriptome, which provided a more comprehensive representation of the virulence repertoire of the species when used as reference for meta-transcriptomic analysis of naturally occurring *Pm. minimum* infections.

## Materials and Methods

### Biological Material

*Phaeoacremonium minimum* strains were purified from *Vitis vinifera* plants (**Supplementary Data S1: Table S1**) as described in Morales-Cruz et al. (2015). For RNAseq, isolates were grown for 28 days in Czapek broth [pH 5.7; (Difco, Detroit, MI, United States)] amended with 0.1% yeast extract (Sigma-Aldrich, Saint-Louis, MO, United States) and 0.1% malt extract (Oxoid Ltd, Basingstoke, United Kingdom) at 25°C in both stationary and rotating (150 rpm) conditions in triplicates. Stationary cultures were kept in complete darkness, while rotating cultures were in ambient light.

### DNA Extraction, Library Preparation, Sequencing, and Assembly

DNA extraction, quality control, and library preparation for PacBio and Illumina sequencing were performed as described in Massonnet et al. (2018) and Morales-Cruz et al. (2015), respectively. SMRTbell libraries were sequenced using 11 cells of a PacBio RSII system (DNA Technologies Core Facility, University of California Davis), which generated 1,110,178 reads with median and maximum lengths of 8.5 and 50 kbp, respectively, for a total of 10.1 Gbp (**Supplementary Data S1: Table S2**). Illumina sequencing was conducted on a HiSeq2500 sequencing platform in 150 paired-end mode (DNA Technologies Core Facility, University of California Davis), yielding 20,231,286 ± 4,530,073 reads per sample (**Supplementary Data S1: Table S3**). For UCR-PA7, raw reads were retrieved from NCBI SRA (SRR654175; Blanco-Ulate et al., 2013).

Contigs were assembled from PacBio reads with HGAP3.0 and error corrected with Quiver (Chin et al., 2013) as described in Massonnet et al. (2018). To estimate error rate, Illumina paired-end reads were mapped using Bowtie2 v.2.2.327 (Langmead and Salzberg, 2012), PCR and optical duplicates were removed with Picard tools v.1.119^1^, and sequence variant identified with UnifiedGenotyper from GATK v.3.3.0 (--ploidy 1 --min_base_quality_score 20; McKenna et al., 2010). Prior to gene prediction, repetitive regions were masked using a combination of *ab initio* and homology-based approaches, as described in Jones et al. (2014). BRAKER prediction was carried out on the soft-masked contigs applying GeneMark-ET with the branch point model. As evidence, we used the paired-end RNAseq reads retrieved from GSE64404 (Morales-Cruz et al., 2015), which were mapped onto the genome assemblies using TopHat v.2.1.0 (Trapnell et al., 2009). Only complete protein-coding sequences (CDS) without internal stop codons were retained. Functional annotations were carried out as described in Massonnet et al. (2018) and using the same parameters as in Morales-Cruz et al. (2015): putative Carbohydrate-Active enZYmes (CAZYmes), peroxidases, cytochromes P450 (P450s), cellular transporters, and genes associated with secondary metabolism, were identified using dbCAN (Yin et al., 2012), fPoxDB (Choi et al., 2014), The Cytochrome P450 Homepage (Nelson, 2009), Transporter Classification Database (Saier et al., 2016), and antiSMASH v.4.0.0 (Weber et al., 2015), respectively.

Illumina reads were trimmed using Trimmomatic v.0.36 (Bolger et al., 2014) with options LEADING:3 TRAILING:3 SLIDINGWINDOW:10:20 MINLEN:20 and assembled with SPAdes v.3.10.1 (Bankevich et al., 2012) with option --careful. For each genotype, *k-mer* lengths delivering the most contiguous and complete assembly where chosen for the final assembly (**Supplementary Data S1: Table S3**). Scaffolds (<1 kbp) and sequences detected as contaminants by seqclean (Haas et al., 2008) were removed. Repeats were masked as described above. Sequences can be retrieved from NCBI (PRJNA421316). Genome sequence of *Pm. minimum* isolate 1119 (Pm1119), gene prediction and annotation, and pan-transcriptome sequence can be found in **Supplementary Data S2**. A genome browser of Pm1119 with all relevant tracks can be accessed at^2^.

### Structural Variation Analysis

Whole genome alignments were performed using NUCmer (MUMmer v3.23; Kurtz et al., 2004). SV features and statistics were obtained using dnadiff (Kurtz et al., 2004) and assemblytics (Nattestad and Schatz, 2016). SV coordinates were extracted using show-diff. For LUMPY v.0.2.13 (Layer et al., 2014) and DELLY2 v.0.7.7 (Rausch et al., 2012), trimmed pair-ended reads were mapped onto Pm1119 using Speedseq v.0.1.2 (Chiang et al., 2015). Only SVs predicted as homozygous alternatives (1/1) in DELLY2 and with at least four supporting reads in LUMPY were retained. SVs that overlapped with sites predicted as variant when Pm1119 reads were mapped onto the Pm1119 reference were removed. SV calls of the three methods were compared using bedtools intersect v2.19.1 (Quinlan and Hall, 2010) with a minimum reciprocal overlap of 90% (English et al., 2015). Complete and partial deletions were confirmed by aligning the candidate SV sequences on the respective genome assemblies using GMAP v.2015-11-20 (Wu and Watanabe, 2005).

### Single Nucleotide Polymorphisms (SNP) Calling and Phylogeny Analysis

Single nucleotide polymorphisms were identified as described above. SNPs were called using the UnifiedGenotyper (GATK v.3.3.0) with the Pm1119 Pacbio assembly as reference. The overall ratio of transition (Tr) over transversion (Tv) mutations was 2.1 ± 0.02. These values are consistent with other studies in fungi (Cantu et al., 2013; Jones et al., 2014) and as expected, are higher than the 0.5 ratio that would be obtained if all substitutions were equally probable. Tr/Tv values were significantly higher in exons (2.7; *P*-value = 9e^-12^) compared to introns (2.0) and intergenic space (1.9; **Supplementary Data S1: Figure S1**), further supporting the accuracy of gene models and variant calls (DePristo et al., 2011). To identify genes under positive selection we applied the procedure described in Cantu et al. (2013). Synthetic sequences incorporating the GATK-detected SNPs were generated using FastaAlternateReferenceMaker of GATK. Orthologous transcripts were then aligned and analyzed using Yn00 (Yang, 2007). Any pair-wise comparisons that yielded a aaa > 1 were classified as under positive selection.

### RNA Extraction, Library Preparation and Sequencing

After 28 days of culture in either stationary or rotating condition, fungal suspensions were vacuum-filtered through 1.6 μm glass microfiber filters (Whatman, Maidstone, United Kingdom) and mycelia were collected in a 2-mL micro-centrifuge tube, then immediately frozen in liquid nitrogen and ground to a powder with a TissueLyser II (Qiagen, Hilden, Germany) at 30 Hz for 30 s. One milliliter of TRIzol reagent (Ambion, Austin, TX, United States) was added to the ground mycelia and extraction of total RNA was performed following the manufacturer’s protocol. RNAseq libraries were prepared using the Illumina TruSeq RNA kit v.2 (Illumina, San Diego, CA, United States) and sequenced on an Illumina HiSeq3000 sequencer (DNA Technologies Core Facility, University of California Davis) in single-end 50-bp mode. Sequences were deposited to Short Read Archive (NCBI; SRA accession: SRP126240; BioProject: PRJNA421316).

### RNAseq, *de novo* Transcriptome Assembly, Identification of Isolate-Specific Transcripts and Construction of a Pan-Transcriptome Reference

Reads were first trimmed using Trimmomatic v.0.36 (Bolger et al., 2014) as described above. For each genotype, *de novo* transcriptome assembly was performed using reads from six RNAseq libraries (three replicates at stationary + three replicates at rotating condition) as input for TRINITY v.2.4.0 (Grabherr et al., 2011). Reconstructed transcripts were then mapped on all genome assemblies using GMAP (Wu and Watanabe, 2005) to determine culture cross-contaminations (**Supplementary Data S1: Table S4**). We detected significant contamination of Pm448 cultures by Pm449. Consequently, the RNAseq data of Pm448 were not included in further analyses. Transcripts were then mapped with GMAP onto the Pm1119 reference genome to identify variable transcripts (**Supplementary Data S1: Table S4**). Transcripts that did not map or that mapped with both coverage and identity ≤80% were considered not present in the reference. Transcripts derived from mitochondrial genes, with internal stop codon(s), without a starting methionine or a stop codon were removed. Transcript redundancies were resolved using the tr2aacds program of EvidentialGene (Gilbert, 2013), which selects from clusters of highly similar contigs the “best” representative transcript based on CDS and protein length. The set of non-redundant transcripts absent in Pm1119 was added to the reference transcriptome to compose the *Pm. minimum* pan-transcriptome. In addition, for each isolate, a private transcriptome was created by removing from the Pm1119 reference transcriptome the transcripts detected as deleted in the isolate and adding the *de novo* assembled complete transcripts detected as not present in Pm1119. Private transcriptomes were then mapped on their own genome assembly using GMAP to determine the genomic coordinates of each transcript (**Supplementary Data S3**). Co-linearity of the protein-coding genes flanking the locus of insertion was used to identify the orthologous coordinates in the Pm1119 reference genome.

Trimmed single-end reads were mapped onto their corresponding private transcriptome using Bowtie2 v.2.2.6 with parameters: -q -end-to-end -sensitive -no-unal. Then, sam2counts.py v.0.91^3^ was used to extract counts of uniquely mapped reads (*Q* > 30). Details on trimming and mapping results are reported in **Supplementary Data S1: Table S5**. The Bioconductor package DESeq2 (Love et al., 2014) was used for read-count normalization and for statistical testing of differential expression. *P*-values were adjusted using the Benjamini–Hochberg method (Benjamini and Hochberg, 1995). Genes with an adjusted *P*-values < 0.05 were defined as differentially expressed (**Supplementary Data S4**).

### Closed-Reference Metatranscriptomics

For meta-transcriptomics, the RNAseq data, retrieved from NCBI SRP092409, consisted of eight libraries from Esca-symptomatic plants, one library from a grapevine with Eutypa dieback-symptoms and one library from a grapevine with no trunk disease symptoms. Reads were quality-trimmed as described above and mapped on three different multi-species reference. All three references included the *V. vinifera* PN40024 line transcriptome (v.V1 from^4^) and the predicted transcriptomes of the nine fungal species most commonly associated with grapevine trunk diseases (Morales-Cruz et al., 2018), but differed in the transcriptome reference for *Pm. minimum*: the three references included either: (i) the transcriptome of UCR-PA7 (Morales-Cruz et al., 2015), (ii) the transcriptome of Pm1119, or (iii) the pan-transcriptome of *Pm. minimum*. Rate of non-specific mapping was evaluated by mapping the six *in vitro* samples of Pm1119 culture onto the meta-reference transcriptome with the *Pm. minimum* pan-transcriptome. Reads mapping onto Pm1119 were randomly subsampled using samtools v.1.3.1 (Li et al., 2009) at the median number of reads mapped on *Pm. minimum* pan-transcriptome by the eight Esca samples. Counts of uniquely mapped reads with a mapping quality *Q* > 30 were extracted as described above and details on trimming and mapping results are reported in **Supplementary Data S5**.

## Results and Discussion

### Assembly of Single Molecule Real-Time Sequencing Reads Generates a Complete and Highly Contiguous Reference Genome for *Pm. minimum*

The first objective of this study was to generate a complete and highly contiguous genome assembly, to serve as reference for the comparative genome analyses described below. The genome of *Pm. minimum* isolate 1119 (Pm1119, henceforth; **Supplementary Data S1: Table S1**) was sequenced using single molecule real-time (SMRT) technology at 213× coverage (**Supplementary Data S1: Table S2**). Sequencing reads were assembled into 25 contigs using HGAP3.0 and error-corrected with Quiver (Chin et al., 2013; **Table 1**): 24 contigs formed the nuclear genome with a total size of 47.3 Mbp, whereas the entire mitochondrial genome was assembled into a single 52.5-kbp contig (**Table 1**). N50 and N90 of the nuclear genome were 5.5 and 4.3 Mbp, respectively, representing a significant improvement in sequence contiguity compared to our previous assembly of isolate UCR-PA7, which was generated using short-read sequencing technology (Blanco-Ulate et al., 2013; **Supplementary Data S1: Figure S2**). To evaluate sequence accuracy, we sequenced at 71× coverage the genome of Pm1119 using an Illumina HiSeq2500 system (**Table 1**). Sequence variant analysis with GATK (McKenna et al., 2010) detected only 20 single nucleotide sites with discordant base calls between the two technologies. If we assume that Illumina short reads are correct, we could conclude that the contigs generated using SMRT sequencing had a sequence accuracy of 99.999957%.

**Table 1:** Statistics of the assembled genomes.

Pm. minimum isolate	Pm1119^∗^	Pm1119^∗∗^	UCR-PA7^∗∗^	Pm1118^∗∗^	Pm448^∗∗^	Pm449^∗∗^
Number of contigs	24	270	255	229	700	61
Total assembly size (Mbp)	47.2	45.5	47.6	45.2	45.0	45.7
Longest contig (Mbp)	8.5	3.6	2.3	2.4	1.5	3.1
Shortest contig (kbp)	17.1	1.0	1.0	1.0	1.0	1.0
N50 (Mbp)	5.5	0.725	0.555	0.647	0.209	1.5
N90 (Mbp)	4.3	0.224	0.139	0.225	0.050	0.391
Average GC content (%)	51.06	49.82	49.54	49.9	50.43	49.99
Total repeats (Mbp)	1.1 (2.31%)	0.734 (1.61%)	0.938 (1.97%)	0.674 (1.49%)	0.527 (1.39%)	0.691 (1.51%)


*^∗^Sequenced with PacBio RSII*.

*^∗∗^Sequenced with Illumina HiSeq2500*.

The number of chromosomes comprising the *Pm. minimum* nuclear genome is still unknown. In order to determine the degree of fragmentation of the assembly, we searched for the presence of telomeric repeats in the terminal contig sequences. Telomeric repeats (5′-TTAGGG-3′; Podlevsky et al., 2008) were found at both ends of four contigs and at one end of six other contigs, suggesting that at least four chromosomes were assembled telomere-to-telomere (**Supplementary Data S1: Figure S3**). Protein-coding genes were detected only on nine of the 24 contigs. These nine contigs also had significantly lower repeat content (1.8% vs. 68.1%, *P-*value = 6.1e^-10^) and short-read mapping coverage (73× vs. 2,552×, *P*-value = 1.3e^-9^; **Supplementary Data S1: Table S6**). Overall, these observations strongly suggest that the 15 remaining contigs are fragments derived from intergenic and repetitive regions of the genome. The nine contigs with protein-coding genes comprised 99.2% of the total assembly, with a total size of 46.9 Mbp, which is slightly larger than the genome size estimated using *k*-mer frequency (45.6 Mbp). Approximately 97% of the Core Eukaryotic Genes (Parra et al., 2009) and 99.9% of the BUSCO orthologous genes (Simão et al., 2015) were found in the assembly, supporting the completeness of the assembled gene space (**Supplementary Data S1: Table S7**). Only 1.1 Mbp (2.31%) of the Pm1119 genome was composed of interspersed repeats and low complexity DNA sequences (**Table 1**), a repeat content comparable with other grapevine trunk pathogens (3.6 ± 2.0%; *P*-value = 0.22), but significantly lower than in other Ascomycete plant pathogens (19.8 ± 24.6%; *P*-value = 0.012; **Supplementary Data S1: Table S8**). Finally, we compared the assembly with contigs of the same isolate sequenced using short-reads and assembled with SPAdes (**Supplementary Data S1: Table S3**; Bankevich et al., 2012). Only 16 indels, each smaller than 500 bp, for a total of 1,528 bp (**Supplementary Data S1: Table S9**), were detected with NUCmer (Kurtz et al., 2004) validating the overall structural accuracy of the assembly.

**FIGURE 2:**
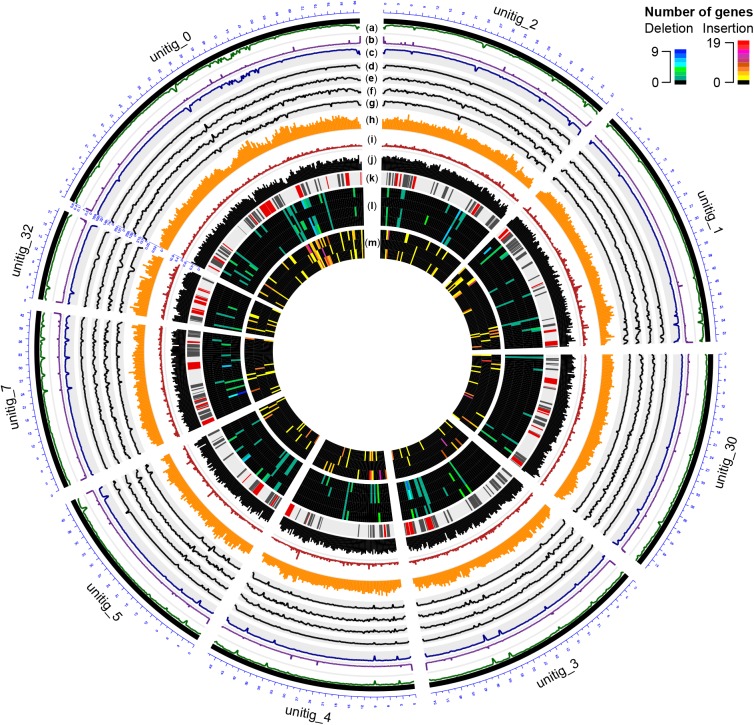
Circular representation of the genome of Pm1119. Different tracks denote: **(a)** percentage of GC content; **(b)** repeat density; **(c–g)** mapping coverage of short Illumina reads: **(c)** Pm1119, **(d)** UCR-PA7, **(e)** Pm1118, **(f)** Pm448, **(g)** Pm449; **(h)** SNP density; **(i)** omega values; **(j)** CDS density; **(k)** BGCs (known classes in red, putative classes in black); **(l)** Number of genes entirely deleted in UCR-PA7, Pm1118, Pm448, or Pm449; **(m)** Number of expressed protein-coding genes inserted in UCR-PA7, Pm1118, or Pm449. Figure was prepared using the OmicCircos Bioconductor package (Hu et al., 2014).

Using BRAKER (Hoff et al., 2015) and RNAseq data as transcriptional evidence, we identified 14,790 protein-coding genes, including 98.05% of the conserved BUSCO orthologs. Gene density was mostly uniform with 3.4 ± 1.0 genes/10 kbp (**Figure 2** and **Supplementary Data S1: Figure S4**), a density comparable to other Ascomycete plant pathogens (Bindschedler et al., 2016). Compared to UCR-PA7, the transcriptome of Pm1119 provided a more comprehensive and accurate representation of the gene space of *Pm. minimum* as shown in **Supplementary Data S1: Figure S5** (**Supplementary Data S1: Table S10**). Over 5,800 more protein-coding genes were detected in Pm1119 than in UCR-PA7 (**Supplementary Data S1: Table S10**) and both alignment coverage and identity were significantly improved when the Pm1119 predicted proteins were aligned to the proteomes of other Ascomycetes (**Supplementary Data S1: Figure S5**).

### Virulence-Factor Focused Annotation Shows Abundant Transport and Secondary Metabolic Functions in the *Pm. minimum* Genome

Annotation focused on processes potentially associated with virulence, such as woody-tissue degradation and colonization, cellular transport and secondary metabolism, as described in Morales-Cruz et al. (2015). We identified a total of 7,699 genes encoding putative virulence factors, corresponding to 52% of *Pm. minimum* predicted transcriptome (**Table 2** and **Supplementary Data S6**). This set of genes comprised 908 Carbohydrate-Active enZYmes (CAZYmes) including 487 cell wall-degrading enzymes (CWDEs) potentially involved in substrate colonization (**Supplementary Data S1: Table S11**). Among the set of putative virulence factors were also 52 peroxidases (including two lignin peroxidases), 157 cytochromes P450 (P450s), 2,742 cellular transporters, and 5,712 genes associated with secondary metabolism.

**Table 2:** Number of genes found for each of the major classes of virulence functions in Pm1119 and in the non-redundant set of variable genes identified in the other isolates (^∗^).

	Pm1119 transcripts	New transcripts ^∗^	Pan-transcriptome
Protein-coding sequences	14,790	455	15,245
Putative virulence factors	7,699	177	7,876
CWDEs	487	3	490
Peroxidases	52	0	52
P450s	157	3	160
Cellular transporters	2,742	44	2,786
Known BGCs^∗∗^	47 (1,739)	15 (43)	48 (1,782)
Putative BGCs^∗∗^	139 (3,973)	35 (107)	139 (4,080)


*^∗∗^The total number of genes in clusters is in parentheses.*

The annotation of Pm1119 in this study is consistent with the previously observed expansion of families of cellular transporters in *Pm. minimum* and confirmed the relatively smaller set of CAZYmes, compared to the dieback-type pathogens examined in our previous analyses (Morales-Cruz et al., 2015). In Pm1119, the Major Facilitator Superfamily (MFS; TCBD code 2.A.1) was the most abundant transporter superfamily, with 816 members and included 200 members of the Sugar Porter (SP) Family (2.A.1.1) and 246 drug-H^+^ antiporter family members [121 DHA1 (2.A.1.2) and 125 DHA2 (2.A.1.3)], which may be involved in toxin secretion (Coleman and Mylonakis, 2009). As observed for other trunk pathogens (Morales-Cruz et al., 2015), the genome of *Pm. minimum* comprised a large number of genes potentially involved in secondary metabolism (5,712 genes). These genes are physically grouped on the *Pm. minimum* chromosomes in 186 biosynthetic gene clusters (BGCs), including 47 belonging to known classes, such as polyketide synthesis (PKS), non-ribosomal peptide synthesis (NRPS), and indole, terpene, and phosphonate synthesis. The identification of a BGC (BGC_137) involved in phosphonate synthesis is noteworthy considering that some phosphonates are known to have antimicrobial properties. Fungi are known to produce these types of compounds (Wassef and Hendrix, 1976), but the key biosynthetic gene in the BGC (phosphoenolpyruvate phosphonomutase, PEP mutase) has been characterized only in bacteria (Yu et al., 2013). Even though one of the predicted proteins of BGC_137 has a putative PEP-mutase domain (BLASTP e-value 6.30e^-60^), until experimentally demonstrated we can only hypothesize that the production of phosphonates may contribute to *Pm. minimum* fitness (Guest and Grant, 1991; Gardner et al., 1992). Nonetheless, in the microbiologically complex environment that *Pm. minimum* inhabits [i.e., in mixed infections with other trunk pathogens and non-pathogenic wood-colonizing fungi (Travadon et al., 2016), in addition to bacteria (Bruez et al., 2015)], it is reasonable to expect this fungus to produce various antimicrobial compounds.

### Comparisons of Multiple Isolates Provides a First Assessment of Structural Variation in the Species and Its Impact on the Gene Space

To investigate the genomic variability in *Pm. minimum*, we sequenced the genomes of four additional isolates from Esca-symptomatic vines (**Figure 1D** and **Supplementary Data S1: Table S1**). Strains isolated from distant geographic locations, with distinct colony morphology and *in vitro* growth rates (**Figure 1D**; **Supplementary Data S1: Figure S6**), were chosen to maximize the potential genetic diversity in the species. An average of 3.4 ± 1.4 Gbp were generated for each isolate, achieving a sequencing coverage of 72 ± 29× (**Supplementary Data S1: Table S3**). Sequencing reads were directly used to identify SNPs. Using GATK, we found a total of 1,389,186 SNPs (**Supplementary Data S1: Table S12**). SNP density was higher in introns (10.8 ± 2.8 SNPs/kbp) compared to exons (4.9 ± 1.5 SNPs/kbp) and intergenic space (8.8 ± 2.4 SNPs/kbp), supporting the overall accuracy of the gene models (**Supplementary Data S1: Figure S1**). Phylogenetic analysis based on the SNPs (**Figure 1D**) indicated that Pm1118 and Pm448 are genetically closer to Pm1119 and Pm449, respectively. SNP information was used to estimate the selective pressure acting on each of the protein-coding genes in the *Pm. minimum* genome using Yn00 (**Figure 2** and **Supplementary Data S6**; Li et al., 1985; Yang, 2007). Interestingly, gene members of the BGCs involved in terpene synthesis were significantly overrepresented (*P*-value = 2.8e^-3^; **Supplementary Data S1: Table S13**) among the 2,136 protein-coding genes with signature of positive selection (aaa > 1). Higher fungi are known to produce a multitude of terpenoid compounds with a wide range of biological functions, such as mycotoxins, antibiotics, and microbial regulators (Collado et al., 2007; Bräse et al., 2009). Signatures of positive selection in the genes involved in terpenoid biosynthesis may suggest that this pathway has played a role in recent adaptation of *Pm. minimum* (Vitti et al., 2013). Signatures of positive selection were found in genes encoding putative virulence factors also in other plant pathogens (Aguileta et al., 2010; Stukenbrock et al., 2011; Hacquard et al., 2012; Cantu et al., 2013; Sharma et al., 2014; Silva et al., 2015), some of which were confirmed experimentally to contribute to virulence (Aguileta et al., 2012; Poppe et al., 2015; Schweizer et al., 2018).

**FIGURE 3:**
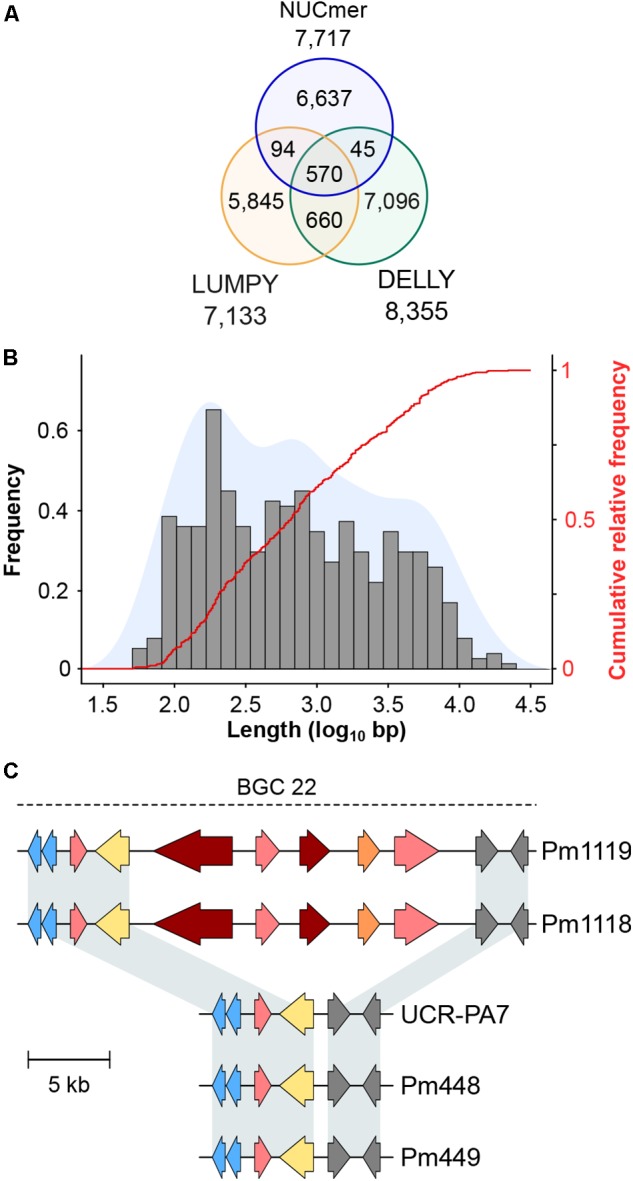
**(A)** Venn diagrams showing the overlap between results of the three methods used to call structural variants (SVs) sites. **(B)** Size distribution of the deleted genomic regions in the four isolates compared to Pm1119. **(C)** Example of a deletion event occurring in UCR-PA7, Pm448, and Pm449 relative to Pm1118 and Pm1119, which affected the composition of a BGC associated with polyketide synthesis. Arrows represent genes coding for core biosynthetic genes (red), additional biosynthetic genes (pink), P450s (blue), cellular transporters (yellow), and FAD-binding proteins (orange). Gray arrows correspond to genes predicted to be part of the biosynthetic gene cluster (BGC), but with other annotations.

To explore genomic structural diversity, we assembled the genomes of the four isolates and compared all assemblies (**Table 1** and **Supplementary Data S1: Table S3**). Total assembly size varied slightly among isolates, from 45 Mbp for Pm448 to 47.6 Mbp for UCR-PA7, and N50 values ranged from 0.2 Mbp for Pm448 to 1.5 Mbp for Pm449. NUCmer analysis of whole-genome alignments (**Supplementary Data S1: Figure S7**) determined that at least 91.9% of the assemblies aligned to Pm1119 (**Supplementary Data S1: Table S14**) and identified multiple insertion/deletion events [≥50 bp/indel; ∼1 Mbp of structural variant sites (SVs) per isolate] in all genotypes relative to Pm1119 (**Supplementary Data S1: Table S9**). Because whole-genome alignments depend on the contiguity and completeness of the sequences, the results of NUCmer may have been confounded by the fragmentation of the isolates that were assembled from short reads (Alkan et al., 2011). We therefore also applied LUMPY (Layer et al., 2014) and DELLY (Rausch et al., 2012), both of which use sequencing read alignment information to identify SVs. Pm1119 was used as reference for both analyses and, therefore, the detected SVs are all relative to Pm1119. LUMPY and DELLY identified 7,133 and 8,355 SVs, respectively. Only 1,233 SVs were identified by both programs. These common SVs included 263 translocations, 861 deletions, 44 duplications, and 65 inversions (**Figure 3A** and **Supplementary Data S7**). Forty six percent of the SVs (570 SVs) identified by both programs were also detected by NUCmer (**Figure 3A**). The limited overlap between results of the three programs confirmed previous reports that showed the importance of using multiple callers to reduce the false discovery rate at the cost of reducing sensitivity of SV detection (Jeffares et al., 2017; Sedlazeck et al., 2017). All but one of the SVs detected by the three programs were deletions (568 ≥ 50 bp SVs; 1.01 Mb total size; **Supplementary Data S7**). All three methods identified one interchromosomal translocation in Pm448, whereas they did not agree on any insertion event relative to Pm1119, demonstrating the difficulty in detecting this type of structural variation. UCR-PA7, Pm448, and Pm449 presented on average 228 ± 1 deletions corresponding to 479 ± 25 kbp (**Table 3**), while Pm1118 showed fewer events (166) for a shorter total length of 256 kbp.

**Table 3:** Size, number, and composition of the structural variants identified when comparing the four *Pm. minimum* isolates with Pm1119.

	UCR-PA7			Pm1118			Pm448			Pm449		
SVs	Total	Del	Ins	Total	Del	Ins	Total	Del	Ins	Total	Del	Ins
Number of SV	308	227	81	211	166	45	227	227	0	311	229	82
Total SV size (kbp)	630.5	471.2	159.3	341.8	256.3	85.4	508.1	508.1	0	599.1	458.5	141.4
Number of genes in SVs	249	86	163	123	30	93	91	91	0	208	80	128
Genes members of BGCs in SVs	98	51	47	48	11	37	53	53	0	93	50	43
BGC members enrichment (*P*-value)	N.S.	0.000069	N.S.	N.S.	N.S.	N.S.	0.000099	0.000099	N.S	N.S.	0.000011	N.S.


*Indel genes belonging to BGCs were tested for overrepresentation using Fisher’s exact test. P-values are indicated. SV, structural variant; Del, deletions; Ins, Insertions; N.S., statistically non-significant.*

**FIGURE 4:**
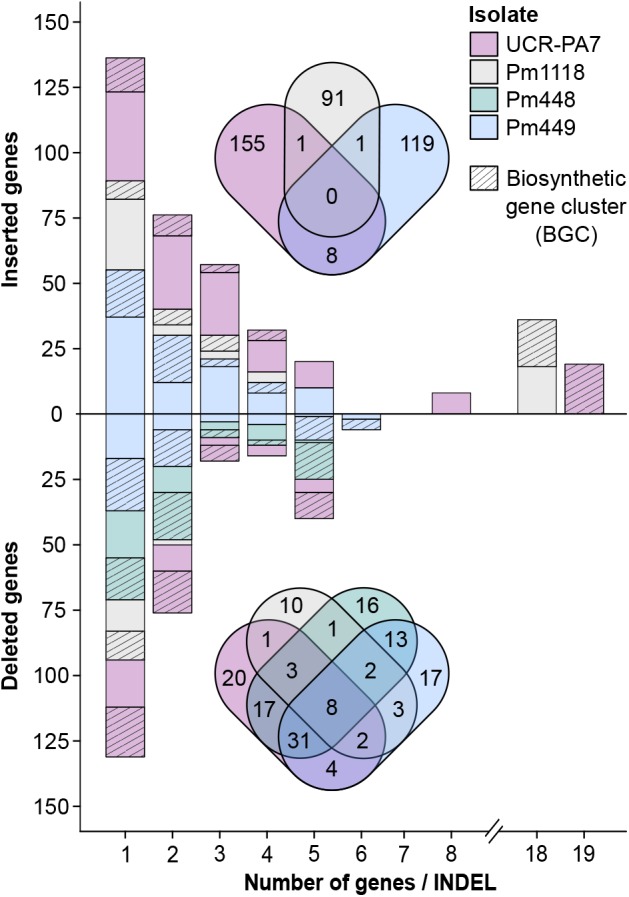
Size and amount of detected indel events encompassing protein-coding genes. The bar plot shows the number of genes and the size of the gene clusters in structural variant sites as well as the proportion of genes associated with BGCs. Venn diagrams show the overlap between genes in structural variant sites detected in the different *Pm. minimum* isolates.

Comparison of deletion events among isolates (**Supplementary Data S1: Figure S8A**) revealed that few events were shared by the four isolates (19/568) and the majority of deletions were isolate-specific (390/568). Pm448 and Pm449 shared almost half of their deletions (105), reflecting their close genetic relationship (**Figure 1D**). The size of deletions ranged from 51 bp to 22 kbp, with a median size of 663 bp (**Figure 3B**). As expected in genomes with a very dense gene space, deletions led to the removal of several protein-coding genes in the four isolates, relative to Pm1119 (**Table 3** and **Figure 4**; **Supplementary Data S1: Figure S8B**). Interestingly, the detected SVs often encompassed regions in the genome encoding putative virulence functions, such as secondary metabolism and cell wall degradation. Entirely-deleted genes in UCR-PA7, Pm448, and Pm449 were significantly enriched in genes belonging to BGCs (*P*-value ≤ 0.01; **Figure 4** and **Supplementary Data S8**). Genes involved in PKS (t1pks) were significantly overrepresented among entirely- and partially-deleted genes in UCR-PA7 and Pm448, whereas two deletion events resulted in the removal of six of the 30 genes belonging to the BGC involved in phosphonate synthesis in Pm449. We also identified a deletion in UCR-PA7, Pm448, and Pm449 that included five adjacent genes all belonging to BGC_22, which is potentially involved in PKS (**Figure 3C**). Polyketides form a large group of biologically active compounds in fungi, including mycotoxins, and antifungal and antibiotic products (Weissman, 2009; Huffman et al., 2010). The extensive diversity within this secondary metabolite group is due to multiple factors that can affect the structure of the synthesized metabolite, such as the number of acyl units assembled by the polyketide synthase and their degree of reduction and *C*-methylation, the type of extender unit used, and the possibility of cyclization of the polyketide chain (Cox and Simpson, 2009). The genes affected by the indel in BGC_22 encode a polyketide synthase and an halogenase (the two core biosynthetic enzymes of the BGC), as well as two *O*-methyltransferases and a FAD-binding monooxygenase, which may be involved in chemical modifications of the final polyketide. Deletions were also enriched (*P*-value ≤ 0.01) in genes involved in cell wall degradation, with the partial removal of two genes encoding enzymes potentially involved in hemicellulose degradation (CE3s; **Supplementary Data S8**).

Because BGCs were overrepresented in the structurally variable sites, we can hypothesize that the acquisition or loss of secondary metabolism functions may have an adaptive effect on fitness in *Pm. minimum*. While the acquisition of BGCs may contribute to virulence or antimicrobial activities (Slot, 2017), the loss of accessory products of the secondary metabolism may be adaptive, for example, by evading recognition of the plant immune system (Raffaele and Kamoun, 2012). Patterns of presence/absence polymorphisms of virulence genes have been identified in other populations of fungal pathogens, mainly those with a biotrophic lifestyle (Gout et al., 2007; Dai et al., 2010; Sharma et al., 2014; Faino et al., 2016; Plissonneau et al., 2016).

### Comparison of *de novo* Assembled Transcriptomes Identifies Additional Indel Events and Variable Genes in *Pm. minimum*

The analysis of structural variation described above failed to identify any insertion event relative to the reference genome. To identify variable genes that are not present in the reference, we therefore used an alternative approach: direct comparisons of protein-CDS of each isolate with the gene space of the reference genome. This approach has previously identified variable genes in plants (Hansey et al., 2012; Hirsch et al., 2014; Jin et al., 2016). Due to the potential bias caused by the fragmentation of the genomic assemblies of the resequenced isolates, we compared the transcriptomes reconstructed by *de novo* assembly of high-coverage RNA sequencing (RNAseq) reads. To maximize the diversity and completeness of the sequenced transcriptomes, all isolates were cultured *in vitro*, to generate a higher transcript coverage compared to *in planta* samples (Massonnet et al., 2018). Both stationary and rotating cultures were used, to increase the number of protein-coding genes expressed under different culture conditions known to affect both fungal growth and gene expression (Feng and Leonard, 1998; Moreno et al., 2007; Ibrahim et al., 2015; **Supplementary Data S1: Table S5**). The transcriptome of each isolate was *de novo* assembled by pooling the reads obtained from three replicates per culture condition. An average of 25,833 ± 5,970 transcripts per isolate were assembled using Trinity (Grabherr et al., 2011; **Supplementary Data S1: Table S4**). The contigs were then mapped on Pm1119 to identify transcripts absent from the reference genome. All of the *de novo* assembled transcripts of Pm1119 mapped onto the Pm1119 genome, thereby confirming the completeness of the gene space in the reference. The transcripts from UCR-PA7, Pm1118 and Pm449 that did not map onto the Pm1119 genome were merged using EvidentialGene (Gilbert, 2013), to generate a non-redundant set of protein-CDS. We identified a total of 455 CDS encoding complete proteins that were not present in the Pm1119 reference: 11 of these were shared by two isolates, whereas 195, 98, and 151 were found only in UCR-PA7, Pm1118, and Pm449, respectively (**Supplementary Data S3** and **Supplementary Data S1: Figure S9**). Predicted proteins of the 455 new transcripts were 349 ± 236 amino acid long, which is comparable to the proteins predicted in Pm1119 (**Supplementary Data S1: Figure S10**). Three of these predicted proteins were annotated as CAZYmes with plant cell wall-degrading functions, three as P450s, 44 as transporters, and 150 as members of BGCs (**Supplementary Data S3**), further supporting the variability between isolates in the content of genes involved in cellular transport and secondary metabolism.

By mapping the 455 CDS on their respective genomes, we identified the coordinates of each insertion relative to Pm1119 (**Supplementary Data S3** and **Table 3**). Many of the insertions involved blocks of multiple genes: 42%, 24%, and 33% were insertions of more than one gene in UCR-PA7, Pm449, and Pm1118, respectively (**Figure 4**). The largest inserted block involved 19 adjacent genes in UCR-PA7. In this isolate, we also identified a single SV that involved a complete BGC associated with terpene synthesis, composed of three adjacent genes encoding a P450, a C6 finger transcription factor, and a terpene cyclase. Interestingly, one third of the indels were flanked at both sides by parts of BGCs, further supporting the hypothesis that BGCs are hotspots for fungal genome evolution (Wisecaver et al., 2014). Variation in secondary metabolite clusters has been intensively studied and characterized between fungal species (Proctor et al., 2013; Wiemann et al., 2013; Cacho et al., 2015; Zhu et al., 2015; Ding et al., 2016); such variation could explain the presence/absence of some metabolites or the difference in the metabolite structure between fungal species (Chooi et al., 2010; Gao et al., 2011; Cacho et al., 2012). Only a few studies have focused on variation of gene content among BGCs within a single species. Intra-species genomic changes, including partial or complete BGC cluster gain and loss, have been observed in the opportunistic human pathogen *Aspergillus fumigatus* (Lind et al., 2017), the plant pathogen *Aspergillus flavus* (Gibbons et al., 2012), and the mycotoxigenic fungi *Aspergillus niger* and *Aspergillus welwitschiae* where this variation impacted the production of fumonisin and ochratoxin (Susca et al., 2016). Copy variation of the entire penicillin BGC has been observed between strains of *Penicillium chrysogenum* (Nijland et al., 2010).

### Analysis of Expression of Structural Variant Gene Clusters Reveals the Impact of Indels on Co-expression of Adjacent Genes

**FIGURE 5:**
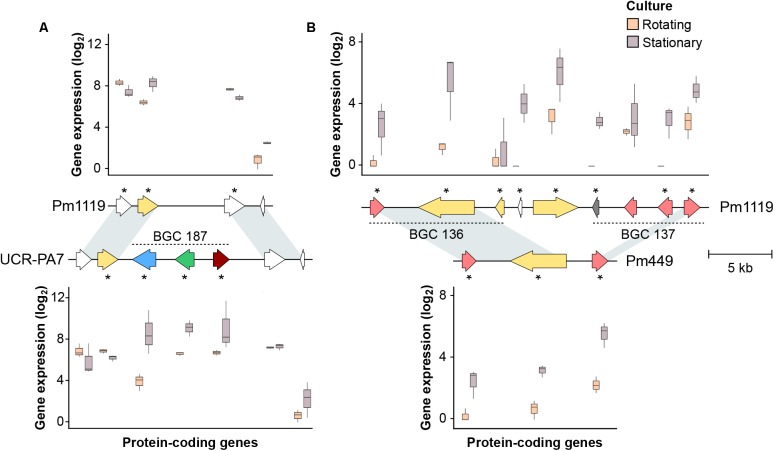
Examples of indels involving co-expressed gene clusters. In **(A)**, the whole co-expressed gene cluster BGC_187 identified in UCR-PA7 is deleted in Pm1119. In **(B)**, the deletion of adjacent co-expressed genes did not alter the co-expression pattern of the genes flanking the structural variant site. Asterisks identify genes significantly differentially expressed *in vitro* between rotating and stationary cultures. Arrows represent genes coding for core biosynthetic genes (red), additional biosynthetic genes (pink), P450s (blue), cellular transporters (yellow), and transcription factors (green). Gray arrows correspond to genes predicted to be part of the BGC, but with other annotations.

Physically clustered genes tend to be co-expressed, due to shared regulatory mechanisms (Lawler et al., 2013; Massonnet et al., 2018). Therefore, to assess the extent of co-expression in the *Pm. minimum* transcriptome and, further, the impact SVs may have on the co-expression of clustered virulence factors, we analyzed the genome-wide patterns of expression dynamics between the two *in vitro* culture conditions (i.e., stationary and rotating cultures) among isolates. RNAseq reads of each isolate were mapped on their respective transcriptomes constructed by combining the shared CDS with Pm1119 and their private *de novo* assembled CDS, as described above. For each isolate, an average of approximately 6 million reads per sample were mapped, detecting an average of 96.5 ± 0.6% of the CDS per isolate (**Supplementary Data S1: Table S5**). Comparison of the detected protein-coding genes between the two culture conditions showed condition-specific gene expression: the expression of 779 ± 227 and 204 ± 161 genes were detected exclusively in stationary and rotating cultures, respectively (**Supplementary Data S1: Figure S11**). Interestingly, the majority of the genes that displayed a condition-specific expression (56.6 ± 2.1%) were associated with secondary metabolism. Condition-specific expression confirmed the importance of using different culture conditions to expand the transcriptional profile of the gene space of *Pm. minimum*. An average of 5,824 ± 2,259 transcripts was detected as differentially expressed between stationary and rotating cultures (adj. *P*-value < 0.05; **Supplementary Data S4** and **Supplementary Data S1: Figure S12**). More than one third of both up- and down-regulated genes were members of BGCs, confirming the effect of the culture condition on fungal secondary metabolism (**Supplementary Data S1: Table S15**; Shih et al., 2007; Ibrahim et al., 2015). Approximately 24% of the differentially expressed genes of each isolate were composed of genomic clusters containing at least three adjacent co-expressed genes (**Supplementary Data S1: Table S16**), confirming that transcriptional modulation in *Pm. minimum* involves groups of physically clustered protein-coding regions, as seen in other trunk pathogens (Massonnet et al., 2018). The analysis also showed the transcriptional modulation of a total of 295 genes involved in SVs (74 ± 40 per isolate). Interestingly, some co-expressed genomic clusters contained genes from the Pm1119 reference genome together with genes present only in specific isolates (**Figure 5**), suggesting that structural variation within co-expressed genomic clusters does not affect the co-regulation of the other BGC members. Co-expression of genes within a cluster can be due to shared regulatory mechanisms, such as transcription factors and chromatin remodeling (Fox and Howlett, 2008; Brakhage, 2013). We can hypothesize that these regulatory functions may not always be affected by a partial deletion of the cluster and, in case of insertion, may contribute to the transcriptional regulations of genes inserted within or close to the cluster. Other studies point to similar results; for example, Bok et al. (2006) showed that a primary metabolism gene was co-expressed with secondary metabolite genes when artificially placed inside the sterigmatocystin cluster in *Aspergillus nidulans*. In addition, some groups of co-expressed genes were composed entirely of genes associated with a single indel. These included the terpene biosynthetic cluster (BGC_187) identified in UCR-PA7 (**Figure 5**).

### The Addition of the Pan-Transcriptome to a Multi-species Reference Expands the Set of Detectable *Pm. minimum* Virulence Activity in Mixed Infections in the Field

We previously showed that by mapping RNAseq reads on a multi-species reference, we can profile within the mixed infections that naturally occur in the field the expression of putative virulence functions of individual fungi (Morales-Cruz et al., 2018). With such a high level of SV involving the gene space and clusters of putative virulence factors, however, we hypothesized that a single genome reference is not sufficient to represent the complete repertoire of virulence functions of *Pm. minimum.* We therefore compiled a transcriptome reference, a pan-transcriptome, which incorporated the variable genes identified in all isolates, i.e., the non-redundant set of CDSs identified in the resequenced isolates. This preliminary pan-transcriptome comprised 14,642 core genes and 603 variable genes. Approximately half of the variable genes were composed of putative virulence factors, mostly associated with secondary metabolism (232 genes) and cellular transport (64 genes). RNAseq data from the same vine samples we previously examined, collected from Esca-symptomatic vines, were mapped on the following: a multi-species transcriptome that included the genome sequence of grape “PN40024,” nine trunk pathogens, and either the CDS of UCR-PA7, the CDS of Pm1119, or the pan-transcriptome of *Pm. minimum*.

**FIGURE 6:**
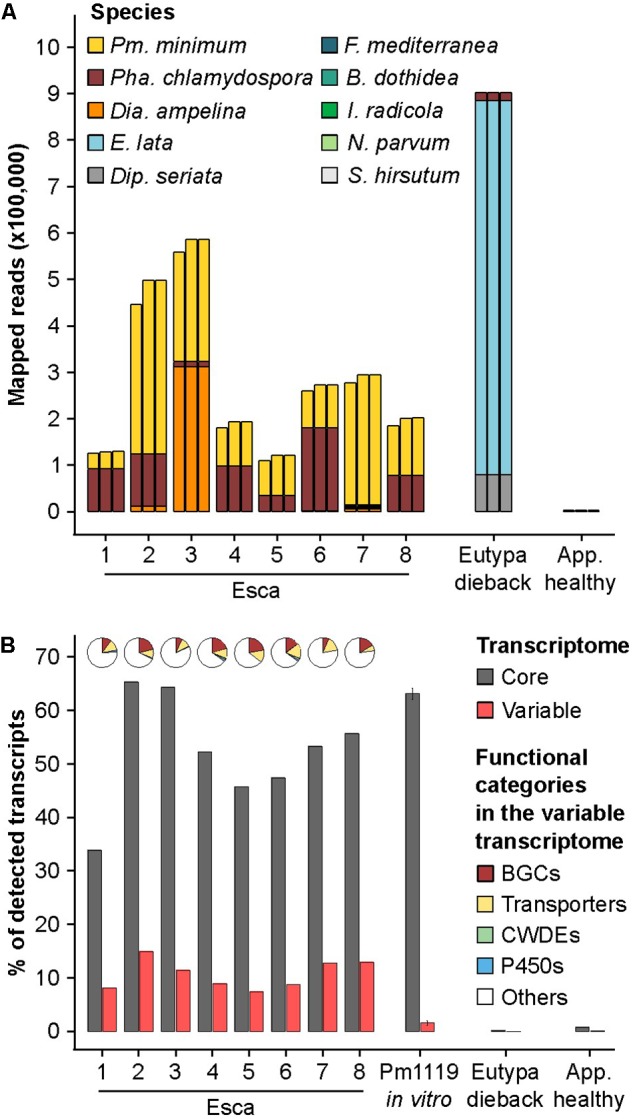
Mapping results of metatranscriptomics data on a multi-species reference including different *Pm. minimum* transcriptome references. **(A)** Stacked bars show the counts of metatransciptomics reads aligned to the transcriptomes of each trunk pathogen included in the multi-species reference using for *Pm. minimum*, from left to right, the transcriptomes of UCR-PA7 (Blanco-Ulate et al., 2013), Pm1119, or the pan-transcriptome. **(B)** Percentage of the core and variable transcripts detected in each sample when using *Pm. minimum* pan-transcriptome in the multi-species reference transcriptome. Pie charts represent the composition of functional categories among the variable transcripts detected in each sample. RNAseq data from Pm1119 *in vitro* cultures were included to determine the level of non-specific mapping on variable genes. BGCs, biosynthetic gene clusters; CWDEs, cell wall-degrading enzymes; P450s, cytochromes P450; *Pm*., *Phaeoacremonium*; *Pha*., *Phaeomoniella*; *Dia*., *Diaporthe*; *E*., *Eutypa*; *Dip*., *Diplodia*; *F*., *Fomitiporia*; *B*. *Botryosphaeria*; *I*., *Ilyonectria*. App. healthy, apparently healthy plant without trunk-disease symptoms.

The inclusion of the Pm1119 reference resulted in an average increase of 13.4% of the number of reads assigned to *Pm. minimum*, compared to UCR-PA7, without affecting the read counts attributed to the other trunk pathogens. This demonstrates the value of a more complete and contiguous genome in transcriptomic studies (**Figure 6A**; **Supplementary Data S5**). The inclusion of the pan-transcriptome led to only a slight increase in total read mapping compared to Pm1119, resulting in the detection of 10.6% of the variable CDS on average per sample (**Figure 6B**). In total, 257 variable transcripts (43% of the variable transcriptome) were detected across the eight vine samples, including 28 transcripts encoding cellular transporters and 94 transcripts associated with secondary metabolism. In all eight samples, transport and secondary metabolism were the most abundant functional categories among the expressed variable transcripts (**Figure 6B**). The detection in natural occurring infections of a large portion of the variable transcriptome, and especially of the secondary metabolism-associated variable transcripts, confirms the validity of incorporating pan-transcriptomes in closed-reference metatranscriptomic studies and further suggests that variable genes may play a role during grapevine infections.

## Conclusion

In this study, we described the genomic diversity among isolates of *Pm. minimum* and showed that detectable structural variation impacted blocks of adjacent virulence genes, preferentially those forming BGCs involved in secondary metabolism. Because in sexually reproducing fungi like *Pm. minimum*, selection pressure is expected to rapidly eliminate deleterious genes or alleles, it is reasonable to hypothesize that the observed structural variation is maintained because it has adaptive effect on fitness. This hypothesis is also supported by the key role that toxins, a product of secondary metabolism, play during plant colonization (Kimura et al., 2001; Andolfi et al., 2011) and interactions with other microorganisms (Braga et al., 2016). However, we cannot rule out the alternative scenario in which variable genes are rare because they have only a marginal deleterious effect on fitness and, therefore, are not easily lost by microbial populations (Vos and Eyre-Walker, 2017). More experiments are required to determine the biological implications of the observed structural variation and understand the role that acquisition or loss of the variable functions play in *Pm. minimum* adaptation and virulence. As sequencing costs continue to decline, we can expect that genome-wide association studies, based on whole-genome resequencing of hundreds of isolates, will help link structural variation to pathogen virulence. With the ability to now genetically transform *Pm. minimum* (Pierron et al., 2015), the addition or deletion of variable genes, combined with the appropriate experiments to assess *Pm. minimum* fitness, will shed light on the evolutionary role played by structural polymorphisms and the associated variable functions.

## Author Contributions

DC and MM conceived the study. KB and PR provided biological material. DL and RT carried out the culture experiments. RF-B and AM-C performed the DNA and RNA extraction, and SMRTbell and RNAseq libraries. MM, AM, and DC carried out the computational analysis. MM and DC wrote the manuscript. All authors read and approved the final manuscript.

## Conflict of Interest Statement

The authors declare that the research was conducted in the absence of any commercial or financial relationships that could be construed as a potential conflict of interest.

## Footnotes

1 http://broadinstitute.github.io/picard/

2 https://cantulab.github.io/data

3 https://github.com/vsbuffalo/sam2counts

4 http://genomes.cribi.unipd.it/grape/

## Supplementary Material

DATA S1 Supplementary tables and figures.

DATA S2 Genome assembly and protein-coding gene coordinates of Pm1119 and pan-transcriptome sequences.

DATA S3 Functional annotation of the 455 transcripts not present in Pm1119 and genomic location of the shared Pm1119 CDS and the private CDS on the genomes of each isolate.

DATA S4 Differentially expressed genes between rotating and stationary culture conditions for each isolate (adj. *P*-value < 0.05).

DATA S5 Result of the metatranscriptomics analysis of field grapevine samples. **(A)** Statistics of raw, trimmed and mapped RNAseq data. **(B)** Number of total reads aligned on each grapevine trunk pathogen species when using as reference the multispecies reference and for *Pm. minimum* either UCR-PA7 (Blanco-Ulate et al., 2013), Pm1119, or the *Pm. minimum* pan-transcriptome. **(C)** Number of detected *Pm. minimum* transcripts. **(D)** List of the 265 *Pm. minimum* variable transcripts detected across the eight Esca-symptomatic plant samples.

DATA S6 Annotations of the Pm1119 predicted protein-coding genes.

DATA S7 Structural variations detected by **(A)** NUCmer, **(B)** DELLY and **(C)** LUMPY, and the overlaps between results of **(D)** NUCmer and DELLY, **(E)** NUCmer and LUMPY, **(F)** DELLY and LUMPY, **(G)** NUCmer, DELLY and LUMPY.

DATA S8 **(A)** Deletion events identified by the three SV-callers **(A)**, genes entirely **(B)** and partially **(C)** deleted and their corresponding enriched functional categories (*P*-value < 0.01; **D** and **E**, respectively).
