# Supplementary material for: Whole-Genome Resequencing and Pan-Transcriptome Reconstruction Highlight the Impact of Genomic Structural Variation on Secondary Metabolite Gene Clusters in the Grapevine Esca Pathogen Phaeoacremonium minimum
Source: Front Microbiol. 2018 Aug 13;9:1784. doi: 10.3389/fmicb.2018.01784 (PMC6099105; doi:10.3389/fmicb.2018.01784)
Supplement: DATA S1 — Supplementary tables and figures. [file Data_Sheet_1.pdf]

Whole-genome resequencing and pan-transcriptome reconstruction highlight the impact of genomic structural variations on secondary metabolite gene clusters in the grapevine Esca pathogen *Phaeoacremonium minimum*

Mélanie Massonnet[1a], Abraham Morales-Cruz[1a], Andrea Minio[1], Rosa Figueroa-Balderas[1], Daniel P. Lawrence[2], Renaud Travadon[2], Philippe E. Rolshausen[3], Kendra Baumgartner[4], and Dario Cantu[1*]

[1] Department of Viticulture and Enology, University of California Davis, Davis CA 95616, USA
[2] Department of Botany and Plant Sciences, University of California Riverside, Riverside CA 92521, USA
[3] Department of Plant Pathology, University of California Davis, Davis CA 95616, USA
[4] United States Department of Agriculture - Agricultural Research Service, Crops Pathology and Genetics Research Unit, Davis CA 95616, USA

[a] Authors contributed equally to this work.

***Correspondence:**
Dario Cantu
dacantu@ucdavis.edu

Data S1

This file contains:
- Tables S1-S16
- Figures S1-S12

Supplementary Tables:

Table S1: *Phaeoacremonium minimum* isolates used in this study and GenBank number of their internal transcribed spacer (ITS) sequence.

Isolate ID	Host	Location	Year	GenBank number
**UCR-PA7**	Thompson Seedless	Fresno County, California	2011	MG149593
**Pm1118**	Syrah174/3309C	Sonoma County, California	2012	MG149591
**Pm1119**	Syrah174/3309C	Sonoma County, California	2012	MG149592
**Pm448**	Niagra	Strafford County, New Hampshire	2008	MG149589
**Pm449**	Cabernet Sauvignon	New York	2008	MG149590

Table S2: Statistics and SRA accession numbers of PacBio sequences.

**Pacbio RSII (P6-C4) reads***	
Total number of sequences	1,110,178
Total length (bp)	10,106,026,198
Average length (bp)	9,103.07 ± 5,985.83
Median length (bp)	8,477
Longest sequence (bp)	50,057
Shortest sequence (bp)	50
N25 (bp)	16,394 (125,112 seqs)
N50 (bp)	12,795 (300,459 seqs)
N75 (bp)	8,817 (534,070 seqs)
Average GC content (%)	48.79 ± 6.17
SRA accession numbers	SRR6353500, SRR6353501, SRR6353502, SRR6353503, SRR6353505, SRR6353514, SRR6353515, SRR6353531, SRR6353532, SRR6353533, SRR6353534

* Numbers refer to post-filtering data used for assembly, i.e. secondary data.

Table S3: Sequencing and genome assembly statistics (data refer to sequencing and assembly of Illumina short reads).

**Isolates**	**UCR-PA7**	**Pm1118**	**Pm1119**	**Pm448**	**Pm449**
**Sequencing features**					
Paired-end reads sequenced	55,894,096	14,169,030	22,795,866	19,518,438	24,441,810
Quality-filtered & trimmed paired reads	53,779,656	13,823,892	22,450,310	19,112,050	24,062,586
Total length (bp)	5,377,965,600	2,073,583,800	3,367,546,500	2,866,807,500	3,609,387,900
Sequencing coverage	113.59	43.80	71.13	60.55	76.23
Mean per-base coverage	19.8	37.2	66.1	50	66.1
SRA accession number	SRR654175	SRR6353537	SRR6353530	SRR6353509	SRR6353510
**Genome assembly**					
k-mer sizes used for assembly	63; 65; 67; 69	75; 79; 81; 83; 85; 87	77; 91; 93; 127	73; 75; 77	107; 117; 127
Total number of contigs	255	229	270	700	61
Total assembly size (bp)	47,592,064	45,172,884	45,540,172	44,986,540	45,741,848
Average length of contigs (bp)	186,636	197,262	168,667	64,266	749,866
Minimum length of contigs (bp)	1,030	1,021	1,001	1,003	1,033
Maximum length of contigs (bp)	2,269,869	2,416,940	3,565,007	1,466,947	3,119,176
N50 (bp)	554,644	647,664	725,336	209,038	1,472,620
N90 (bp)	139,073	225,518	224,125	49,501	391,145
Average GC content (%)	49.54	49.90	49.82	50.43	49.99
**Repeats (bp)**					
Total interspersed repeats	583,669	342,275	384,394	312,754	356,222
Simple repeats	312,657	293,789	308,308	277,682	296,255
Low complexity	32,162	28,860	31,946	26,775	29,498
Total repeats (% of genome)	938,477 (1.97)	674,246 (1.49)	733,652 (1.61)	527,045 (1.39)	690,794 (1.51)

Table S4: Statistics of the *de novo* assembled transcriptomes of the five *Pm. minimum* strains. cov, coverage; id, identity.

	**UCR-PA7**	**Pm1118**	**Pm448***	**Pm449**	**Pm1119**
**Reconstructed transcripts**	23,011	36,502	22,836	23,358	23,456
**Mapped on own Illumina genome assembly (cov ≥ 98%, id=100%)**	19,968	17,575	3,864	20,140	20,067
**Mapped on Pm1119 Pacbio genome**	19,181	17,240	N/A	19,516	20,054
Mapped on unique position (cov ≥ 80%, id ≥ 80%)	19,156	17,225	N/A	19,491	20,043
Mapped on multiple positions (cov ≥ 80%, id ≥ 80%) in at least one position	25	15	N/A	25	11
**Isolate-specific transcripts**	785	335	N/A	624	12
Mapped on unique position (cov ≤ 80%, id ≤ 80%)	111	35	N/A	95	0
Mapped on multiple positions (cov ≤ 80%, id ≤ 80%) for all positions	21	7	N/A	22	0
Translocated	26	11	N/A	25	0
Unmapped (no position found)	627	282	N/A	482	12
**Isolate-specific transcripts encoding complete proteins**	205	101	N/A	160	0
Mapped at same loci than Pm1119 genes	40	7	N/A	29	0
Insertions (mapped at others genomic loci than Pm1119 gene)	163	93	N/A	128	0
Insertions having alternative transcripts	2	1	N/A	3	0

*Pm448 RNAseq data were discarded due to cross-contamination.

Table S5: Summary of RNAseq data of the five *Pm. minimum* isolates cultured *in vitro*.

**Isolate**	**Treatment**	**Biological replicate**	**Raw reads**	**Quality-filtered & trimmed reads**	**%**	**Unambiguously mapped**	**%**
Pm449	Rotating	1	15,129,608	15,127,948	100.0	6,657,723	44.0
Pm449	Rotating	2	12,938,295	12,936,930	100.0	6,444,657	49.8
Pm449	Rotating	3	11,521,710	11,520,318	100.0	6,020,411	52.3
Pm449	Stationary	1	14,887,759	14,885,958	100.0	7,173,655	48.2
Pm449	Stationary	2	15,692,980	15,691,213	100.0	6,948,497	44.3
Pm449	Stationary	3	13,641,782	13,640,208	100.0	8,053,323	59.0
Pm448	Rotating	1	17,248,049	17,246,160	100.0	N/A	N/A
Pm448	Rotating	2	12,848,987	12,847,453	100.0	N/A	N/A
Pm448	Rotating	3	14,943,516	14,941,959	100.0	N/A	N/A
Pm448	Stationary	1	12,376,925	12,375,497	100.0	N/A	N/A
Pm448	Stationary	2	12,145,964	12,144,497	100.0	N/A	N/A
Pm448	Stationary	3	11,784,393	11,783,048	100.0	N/A	N/A
Pm1119	Rotating	1	12,525,180	12,523,944	100.0	6,618,666	52.8
Pm1119	Rotating	2	11,950,396	11,949,042	100.0	5,869,452	49.1
Pm1119	Rotating	3	11,975,503	11,974,105	100.0	6,790,114	56.7
Pm1119	Stationary	1	12,324,377	12,323,093	100.0	6,423,504	52.1
Pm1119	Stationary	2	12,568,770	12,567,391	100.0	7,078,544	56.3
Pm1119	Stationary	3	14,233,851	14,232,570	100.0	6,521,113	45.8
Pm1118	Rotating	1	11,338,055	11,336,865	100.0	1,225,639	10.8
Pm1118	Rotating	2	15,095,904	15,094,228	100.0	7,555,545	50.1
Pm1118	Rotating	3	13,891,833	13,890,338	100.0	1,150,805	8.3
Pm1118	Stationary	1	14,801,556	14,800,022	100.0	2,773,030	18.7
Pm1118	Stationary	2	11,797,926	11,796,658	100.0	7,092,301	60.1
Pm1118	Stationary	3	12,532,240	12,530,668	100.0	5,136,684	41.0
UCR-PA7	Rotating	1	13,934,049	13,932,424	100.0	5,026,643	36.1
UCR-PA7	Rotating	2	15,967,943	15,965,921	100.0	6,318,459	39.6
UCR-PA7	Rotating	3	14,608,047	14,606,068	100.0	5,259,023	36.0
UCR-PA7	Stationary	1	13,487,426	13,485,749	100.0	7,828,687	58.1
UCR-PA7	Stationary	2	12,633,500	12,631,721	100.0	6,808,053	53.9
UCR-PA7	Stationary	3	13,365,293	13,363,850	100.0	5,498,546	41.1

Table S6: Analysis of sequencing coverage and interspersed repeat content of the 24 contigs of the Pm1119 reference genome.

Contig_ID	Contig length (bp)	Mapped reads	Coverage	Repeat length (bp)	%
**unitig_0***	8,540,616	4,001,090	70.27	175,859	2.06
**unitig_1***	5,859,229	2,815,606	72.08	93,275	1.59
**unitig_2***	5,712,395	2,726,027	71.58	107,138	1.88
**unitig_3***	5,520,702	2,634,183	71.57	92,611	1.68
**unitig_30***	5,355,821	2,718,740	76.14	88,883	1.66
**unitig_4***	5,008,684	2,417,916	72.41	85,000	1.70
**unitig_5***	4,470,411	2,138,764	71.76	79,681	1.78
**unitig_7***	4,271,344	2,028,817	71.25	75,479	1.77
**unitig_32***	2,189,992	1,160,176	79.46	41,164	1.88
**unitig_34**	37,782	681,154	2,704.28	27,591	73.03
**unitig_12**	34,989	622,944	2,670.60	24,914	71.21
**unitig_33**	30,222	534,074	2,650.75	21,809	72.16
**unitig_38**	27,380	489,988	2,684.38	20,197	73.77
**unitig_37**	26,290	469,036	2,676.13	18,234	69.36
**unitig_36**	24,157	445,743	2,767.79	17,963	74.36
**unitig_35**	23,068	414,253	2,693.69	16,839	73.00
**unitig_9**	22,825	410,966	2,700.76	16,493	72.26
**unitig_14**	22,084	410,898	2,790.92	16,250	73.58
**unitig_8**	21,518	380,537	2,652.69	15,482	71.95
**unitig_40**	20,191	361,893	2,688.52	15,445	76.49
**unitig_13**	20,189	358,194	2,661.31	14,188	70.28
**unitig_6**	18,110	10,628	88.03	1,062	5.86
**unitig_31**	18,090	344,531	2,856.81	12,442	68.78
**unitig_15**	17,102	341,773	2,997.66	12,975	75.87

* The longest 9 contigs accounted for 99.2 % of the total genome assembly and contained 100% of the predicted CDS.

Table S7: Gene space completeness estimation of the Pm1119 reference genome using CEGMA (Parra *et al.*, 2009) and BUSCO (Simão *et al.*, 2015).

**CEGMA**	
Complete CEGs	234 (94.35%)
Partial CEGs	6 (2.41%)
Missing CEGs	8 (3.22%)
**BUSCO**	
Complete Single-Copy BUSCOs	1,411 (98.12%)
Fragmented BUSCOs	15 (1.04%)
Missing BUSCOs	12 (0.01%)

Table S8: Repeat content comparison with other plant pathogen genomes.

Species	Assembly size (Mb)	% Repeat content	Reference
**Grapevine trunk pathogenes**			
Phaeomoniella chlamydospora (UCR-PC4)	27.5	1.84	Morales-Cruz *et al*. (2015)
Diaporthe ampelina (DA912)	47.4	1.85	Morales-Cruz *et al*. (2015)
Phaeoacremonium minimum (Pm1119)	46.9	2.3	This study
Diplodia seriata (DS831)	37.1	2.9	Morales-Cruz *et al*. (2015)
Eutypa lata (UCR-EL1)	54	5.13	Blanco-Ulate *et al*. (2013)
Neofusicoccum parvum (UCD646So)	42.6	6.2	Massonnet *et al*. (2018)
**Ascomycete plant pathogens**			
Fusarium graminearum (PH-1)	36	<3	Möller and Stukenbrok (2017)
Ophiostoma novo-ulmi (H327)	32	3.4	Möller and Stukenbrok (2017)
Leptosphaeria biglobosa 'canadensis' (J154)	32	3.9	Möller and Stukenbrok (2017)
Botrytis cinerea	39	4	Raffaele and Kamoun et al. (2012)
Fusarium solani/*Nectria haematococca* MPVI (77-13-4)	54	<5	Möller and Stukenbrok (2017)
Stagonospora nodorum (SN15)	37	5	Raffaele and Kamoun *et al*. (2012)
Sclerotinia sclerotiorum (1980 UF-70)	38	8	Raffaele and Kamoun *et al*. (2012)
Fusarium verticillioides (7600)	42	8	Raffaele and Kamoun *et al*. (2012)
Magnaporthe oryzae (70–15)	41	10	Möller and Stukenbrok (2017)
Verticillium dahliae (VdLs17)	37	12	Möller and Stukenbrok (2017)
Mycosphaerella graminicola	32-40	18	Raffaele and Kamoun *et al*. (2012)
Zymoseptoria tritici (IPO323)	40	18.6	Möller and Stukenbrok (2017)
Fusarium oxysporum f. sp. *lycopersici* (4287)	60	28	Möller and Stukenbrok (2017)
Leptosphaeria maculans 'brassicae' (v23.1.3)	45	35.5	Möller and Stukenbrok (2017)
Blumeria graminis f. sp. *hordei* (DH14)	120	64	Möller and Stukenbrok (2017)
Blumeria graminis f. sp. *tritici* (96224)	180	90	Möller and Stukenbrok (2017)

Table S9: Structural variants between the resequenced genomes and Pm1119 reference genome identified using NUCmer (Kurtz *et al*., 2004) and Assemblytics (Nattestad and Schatz, 2016).

Query	UCR-PA7		448		449		1118		1119	
**Insertion**										
**Size range**	**Count**	**Total size (bp)**	**Count**	**Total size (bp)**	**Count**	**Total size (bp)**	**Count**	**Total size (bp)**	**Count**	**Total size (bp)**
1-10 bp	20,956	46,611	21,026	47,110	21,052	46,981	13,657	30,186	1,240	1,377
10-50 bp	1,677	29,406	1,771	31,737	1,711	29,916	1,204	21,462	39	938
50-500 bp	166	27,408	248	32,772	172	26,997	150	21,675	9	650
500-10000 bp	76	192,652	61	132,029	95	301,027	52	101,225	0	0
10000-50000 bp	10	138,135	3	40,357	10	136,769	6	90,411	0	0
50000-100000 bp	0	0	0	0	0	0	0	0	0	0
Total	22,885	434,212	23,109	284,005	23,040	541,690	15,069	264,959	1,288	2,965
Total > 50 bp	252	358,195	312	205,158	277	464,793	208	213,311	9	650
**Deletion**										
**Size range**	**Count**	**Total size (bp)**	**Count**	**Total size (bp)**	**Count**	**Total size (bp)**	**Count**	**Total size (bp)**	**Count**	**Total size (bp)**
1-10 bp	20,200	45,577	20,113	45,101	19,855	44,597	12,621	29,244	125	263
10-50 bp	1,744	30,480	1,774	30,830	1,768	30,835	1,128	19,787	125	263
50-500 bp	189	30,899	168	25,630	171	28,527	113	19,151	22	444
500-10000 bp	90	290,958	88	284,703	91	278,585	69	201,493	0	0
10000-50000 bp	19	374,826	16	302,548	18	353,315	15	291,165	0	0
50000-100000 bp	1	52,908	1	52,908	1	62,127	1	62,127	0	0
Total	22,243	825,648	22,160	741,720	21,904	797,986	13,947	622,967	0	0
Total > 50 bp	299	749,591	273	665,789	281	722,554	198	573,936	147	707
**Tandem expansion**										
**Size range**	**Count**	**Total size (bp)**	**Count**	**Total size (bp)**	**Count**	**Total size (bp)**	**Count**	**Total size (bp)**	**Count**	**Total size (bp)**
1-10 bp	1	9	0	0	1	3	2	6	3	19
10-50 bp	0	0	0	0	0	0	3	98	5	83
50-500 bp	7	797	24	3,748	6	682	21	2,692	3	589
500-10000 bp	2	6,848	0	0	1	8,323	0	0	0	0
10000-50000 bp	2	34,685	1	35,450	2	65,618	2	46,152	0	0
50000-100000 bp	0	0	0	0	0	0	0	0	0	0
Total	12	42,339	25	39,198	10	74,626	28	48,948	11	691
Total > 50 bp	11	42,330	25	39,198	9	74,623	23	48,844	3	589
**Tandem contraction**										
**Size range**	**Count**	**Total size (bp)**	**Count**	**Total size (bp)**	**Count**	**Total size (bp)**	**Count**	**Total size (bp)**	**Count**	**Total size (bp)**

1-10 bp	0	0	0	0	0	0	0	0	4	25
10-50 bp	0	0	0	0	0	0	0	0	9	206
50-500 bp	2	368	1	434	3	222	3	553	3	237
500-10000 bp	0	0	0	0	1	7,375	0	0	0	0
10000-50000 bp	1	10,488	0	0	0	0	0	0	0	0
50000-100000 bp	0	0	0	0	0	0	0	0	0	0
Total	3	10,856	1	434	4	7,597	3	553	16	468
Total > 50 bp	3	10,856	1	434	4	7,597	3	553	3	237
**Total**										
Total for all variants	45,390	2,410,391	45,556	1,836,847	45,229	2,835,982	29,201	1,719,885	1,463	4,883
Total for all structural variants > 50 bp	777	2,257,514	800	1,680,767	812	2,682,990	568	1,618,783	16	1,528
Total for all structural variants excluding repeat variation	565	1,160,972	611	910,579	571	1,269,567	432	836,644	15	1,476

Table S10: Comparison of the proteomes predicted in the Pm1119 reference genome and UCR-PA7 (Morales-Cruz *et al*., 2015).

	***Pm. minimum* UCR-PA7**	***Pm. minimum* 1119**
**N. of predicted peptides**	11,591	14,790
**Total peptide length (a.a.)**	4,998,457	7,399,304
**Mean peptide length (a.a.)**	431.23	500.29
**Median peptide length (a.a.)**	368	431

Table S11: *Pm. minimum* CAZyme families involved in plant cell wall degradation.

**Substrate**	**CAZyme family**	**Enzyme activity**	**References**
**Cellulose**	GH5	Endo-β-1,4-glucanase (EC 3.2.1.4)	van Peij *et al.*, 1998
	GH7	Endo-β-1,4-glucanase (EC 3.2.1.4)	Kubicek *et al.*, 2014
	GH12	Endo-β-1,4-glucanase (EC 3.2.1.4)	van Peij *et al.*, 1998
	GH45	Endo-β-1,4-glucanase (EC 3.2.1.4)	Kubicek *et al.*, 2014
	GH6	Cellobiohydrolase (EC 3.2.1.91)	Kubicek *et al.*, 2014
	GH7	reducing end-acting cellobiohydrolase (EC 3.2.1.176)	Gielkens *et al.*, 1999
	GH1	β-glucosidase (EC 3.2.1.21)	
	GH3	β-glucosidase (EC 3.2.1.21)	Dan *et al.*, 2000; Pel *et al.*, 2007
	AA9 (formerly GH61)	Lytic polysaccharide monooxygenase	Beeson *et al.*, 2012; Quinlan *et al.*, 2011
	AA10 (formerly CBM33)	Lytic polysaccharide monooxygenase	
**Hemicellulose**			
Xyloglucan backbone	GH5	Xyloglucan endo-β-1,4-glucanase (EC 3.2.1.151)	Kubicek *et al.*, 2014
	GH12	Xyloglucan endo-β-1,4-glucanase (EC 3.2.1.151)	van Peij *et al.*, 1998; Kubicek *et al.*, 2014
	GH16	Xyloglucan endo-β-1,4-glucanase (EC 3.2.1.151)	Kubicek *et al.*, 2014
	GH44	Xyloglucan endo-β-1,4-glucanase (EC 3.2.1.151)	Kubicek *et al.*, 2014
	GH74	Xyloglucan endo-β-1,4-glucanase (EC 3.2.1.151)	Kubicek *et al.*, 2014
Xylan backbone	GH5	Endo-1,4-β-xylanase (EC 3.2.1.8)	Krengel and Dijkstra, 1996
	GH7	Endo-1,4-β-xylanase (EC 3.2.1.8)	Cantarel *et al.*, 2009
	GH8	Endo-1,4-β-xylanase (EC 3.2.1.8)	Krengel and Dijkstra, 1996
	GH10	Endo-1,4-β-xylanase (EC 3.2.1.8)	Krengel and Dijkstra, 1996
	GH11	Endo-1,4-β-xylanase (EC 3.2.1.8)	Levasseur *et al.*, 2005
	GH30	Endo-1,4-β-xylanase (EC 3.2.1.8)	Krengel and Dijkstra, 1996
	GH43	Endo-1,4-β-xylanase (EC 3.2.1.8)	Krengel and Dijkstra, 1996
	GH3	Endo-1,4-β-xylosidase (EC 3.2.1.37)	van Peij *et al.*, 1997
	GH39	Endo-1,4-β-xylosidase (EC 3.2.1.37)	van Peij *et al.*, 1997
	GH43	Endo-1,4-β-xylosidase (EC 3.2.1.37)	van Peij *et al.*, 1997
	GH52	Endo-1,4-β-xylosidase (EC 3.2.1.37)	van Peij *et al.*, 1997
	GH54	Endo-1,4-β-xylosidase (EC 3.2.1.37)	van Peij *et al.*, 1997
Galacto(gluco)mannan backbone	GH5	β-1,4-endomannanase (EC 3.2.1.78)	Ademark *et al.*, 1998
	GH26	β-1,4-endomannanase (EC 3.2.1.78)	
	GH1	β-1,4-mannosidase (EC 3.2.1.25)	Ademark *et al.*, 2001

	GH2	β-1,4-mannosidase (EC 3.2.1.25)	Ademark *et al.*, 2001
	GH5	β-1,4-mannosidase (EC 3.2.1.25)	Ademark *et al.*, 2001
**Side-chains**	GH2	β-1,4-galactosidase (EC 3.2.1.23)	den Herder *et al.*, 1992; de Vries *et al.*, 1999
	GH35	β-1,4-galactosidase (EC 3.2.1.23)	den Herder *et al.*, 1992; Kumar *et al.*, 1992; de Vries *et al.*, 1999
	GH4	α-galactosidase (EC 3.2.1.22)	
	GH27	α-galactosidase (EC 3.2.1.22)	den Herder *et al.*, 1992; de Vries *et al.*, 1999
	GH36	α-galactosidase (EC 3.2.1.22)	Ademark *et al.*, 2001
	GH57	α-galactosidase (EC 3.2.1.22)	den Herder *et al.*, 1992
	GH3	α-L-arabinofuranosidase (EC 3.2.1.55)	Flipphi *et al.*, 1993a
	GH43	α-L-arabinofuranosidase (EC 3.2.1.55)	
	GH51	α-L-arabinofuranosidase (EC 3.2.1.55)	Flipphi *et al.*, 1993a
	GH54	α-L-arabinofuranosidase (EC 3.2.1.55)	
	GH62	α-L-arabinofuranosidase (EC 3.2.1.55)	
	GH1	β-glucosidase (EC 3.2.1.21)	
	GH3	β-glucosidase (EC 3.2.1.21)	
	GH67	Xylan α-1,2-glucuronidase (3.2.1.131)	
	GH115	Xylan α-1,2-glucuronidase (3.2.1.131)	
	GH29	α-L-fucosidase (EC 3.2.1.51)	
	GH95	α-L-fucosidase (EC 3.2.1.51)	
	GH62	Arabinoxylan arabinofuranohydrolase (EC 3.2.1.55)	Gielkens *et al.*, 1997
	GH31	α-xylosidase (EC 3.2.1.177)	
	GH43	Endo-α-1,5-arabinase (3.2.1.99)	
	GH67	α-Glucuronidase (3.2.1.139)	
	GH53	Endo-β-1,4-galactanase (EC 3.2.1.89)	
	CE1	Acetyl xylan esterase (EC 3.1.1.72)	van Peij *et al.*, 1998
	CE2	Acetyl xylan esterase (EC 3.1.1.72)	van Peij *et al.*, 1998
	CE3	Acetyl xylan esterase (EC 3.1.1.72)	van Peij *et al.*, 1998
	CE4	Acetyl xylan esterase (EC 3.1.1.72)	van Peij *et al.*, 1998
	CE5	Acetyl xylan esterase (EC 3.1.1.72)	van Peij *et al.*, 1998
	CE6	Acetyl xylan esterase (EC 3.1.1.72)	van Peij *et al.*, 1998
	CE7	Acetyl xylan esterase (EC 3.1.1.72)	van Peij *et al.*, 1998
	CE1	Cinnamoyl esterase (EC 3.1.1.-); feruloyl esterase (EC 3.1.1.73)	de Vries *et al.*, 1997; de Vries *et al.*, 2002c
**Pectin**			
Homogalacturonan backbone (1→4)-α-D-galacturonan	PL1	Pectin lyase (EC 4.2.2.10)	Gysler *et al.*, 1990; Harmsen *et al.*, 1990; Kusters-van Someren *et al.*, 1992; de Vries *et al.*, 2002a
	PL1	Pectate lyase (EC 4.2.2.2)	Benen *et al.*, 2000
	PL3	Pectate lyase (EC 4.2.2.2)	Kubicek *et al.*, 2014

	PL9	Pectate lyase (EC 4.2.2.2)	
	GH28	Endo-/exo- polygalacturonase (EC 3.2.1.15)	Bussink *et al.*, 1992; Kester and Visser, 1990; Martens-Uzunova *et al.*, 2006; Parenicova *et al.*, 1998; Parenicova *et al.*, 2000a; Parenicova *et al.*, 2000b
	CE8	Pectin methylesterase (EC 3.1.1.11)	Khanh *et al.*, 1991
Rhamnogalacturonan I (RG-I) backbone	GH28	Endo-/exo- rhamnogalacturonase (EC 3.2.1.171)	Martens-Uzunova *et al.*, 2006; Suykerbuyk *et al.*, 1997
	GH78	α-L-rhamnosidase (EC 3.2.1.40)	
	GH88	D-4,5-unsaturated β-glucuronyl hydrolase (EC 3.2.1.-)	
	GH105	Unsaturated rhamnogalacturonyl hydrolase (EC 3.2.1.172)	Martens-Uzunova and Schaap, 2009
	CE12	Rhamnogalacturonan acetylesterase (EC 3.1.1.-); Pectin acetylesterase (EC 3.1.1.-)	Martens-Uzunova and Schaap, 2009; de Vries *et al.*, 2000
	PL4	Rhamnogalacturonan lyase (EC 4.2.2.-)	de Vries *et al.*, 2002a
	PL11	Rhamnogalacturonan endolyase (EC 4.2.2.23)	
Xylogalacturonan backbone	GH28	Endo-xylogalacturonan hydrolase (EC 3.2.1.-)	van der Vlugt-Bergmans *et al.*, 2000
**Side-chains**	GH3	α-L-arabinofuranosidase (EC 3.2.1.55)	
	GH43	α-L-arabinofuranosidase (EC 3.2.1.55)	Martens-Uzunova and Schaap, 2009
	GH51	α-L-arabinofuranosidase (EC 3.2.1.55)	Martens-Uzunova and Schaap, 2009
	GH2	β-1,4-galactosidase (EC 3.2.1.23)	
	GH35	β-1,4-galactosidase (EC 3.2.1.23)	Martens-Uzunova and Schaap, 2009
	GH3	Endo-1,4-β-xylosidase (EC 3.2.1.37)	
	GH39	Endo-1,4-β-xylosidase (EC 3.2.1.37)	
	GH43	Endo-1,4-β-xylosidase (EC 3.2.1.37)	
	GH43	Endo-1,5-α-L-arabinanase (EC 3.2.1.99)	Flipphi *et al.*, 1993b
	GH93	Exo-α-L-1,5-arabinanase (EC 3.2.1.-)	
	GH53	Endo-β-1,4-galactanase (EC 3.2.1.89)	Martens-Uzunova and Schaap, 2009; de Vries *et al.*, 2002b
	GH127	β-L-arabinofuranosidase (EC 3.2.1.185)	
	CE1	Feruloyl esterase (EC 3.1.1.73)	
**Lignin**	AA2	Manganese peroxidase (EC 1.11.1.13);	
		versatile peroxidase (EC 1.11.1.16);	
		lignin peroxidase (EC 1.11.1.14); peroxidase (EC 1.11.1.-)	
**Auxiliary enzymes**	AA1	Laccase/p-diphenol:oxygen oxidoreductase/ferroxidase (EC 1.10.3.2); ferroxidase (EC 1.10.3.-); laccase-like multicopper oxidase (EC 1.10.3.-)	
	AA3	GMC oxidoreductase	
	AA4	Vanillyl alcohol oxidase (EC 1.1.3.38)	
	AA5	Radical-copper oxidase	

	AA6	1,4-Benzoquinone reductase (EC. 1.6.5.6)	
	AA7	Glucooligosaccharide oxidase (EC 1.1.3.-)	
	AA8	Iron reductase domain	

Table S12: Single-nucleotide polymorphisms (SNPs) identified between the Pm1119 reference genome and the other isolates.

	**SNPs**	**SNPs / kbp**	**Identity (%)**
**Pm1119 *vs.* Pm1119***	20	0.00	99.99995771
**UCR-PA7 *vs.* Pm1119**	410,684	8.68	99.13162129
**Pm1118 *vs.* Pm1119**	210,811	4.46	99.55424661
**Pm448 *vs.* Pm1119**	330,950	7.00	99.30021639
**Pm449 *vs.* Pm1119**	436,741	9.23	99.07652457

*Pm1119 Illumina *vs.* Pm1119 Pacbio

Table S13: Functional category enrichment analysis of the 2,136 protein-coding genes under putative positive selection ($\omega > 1$).

**Functional category**	***P*-value**	**Count in category**	**Total number of genes in category in genome**
BGC_48	$2.22e^{-6}$	10	14
BGC_57	$5.89e^{-5}$	9	15
BGC_152	$3.86e^{-4}$	13	33
BGCs involved in terpene synthesis	$2.84e^{-3}$	31	130
BGC_32	$7.80e^{-3}$	14	49
BGC_36	$8.22e^{-3}$	11	35
The P-type ATPase (P-ATPase) Superfamily	$9.47e^{-3}$	10	31

Table S14: Genome-wide alignments results between all isolates and the Pm1119 reference genome. Analysis was carried out with NUCmer.

	**UCR-PA7**	**Pm448**	**Pm449**	**Pm1118**	**Pm1119**
Number of contigs with at least one alignment	248 (97.25%)	649 (92.71%)	61 (100.00%)	226 (98.69%)	267 (98.89%)
Number of contigs with no alignment	7 (2.75%)	51 (7.29%)	0 (0.00%)	3 (1.31%)	3 (1.11%)
Total number of aligned bases (bp)	45,010,024 (94.57%)	43,348,848 (96.36%)	44,046,558 (96.29%)	43,946,176 (97.28%)	45,499,454 (99.91%)
Total number of unaligned bases (bp)	2,582,040 (5.43%)	1,637,692 (3.64%)	1,695,290 (3.71%)	1,226,708 (2.72%)	40,718 (0.09%)
Total length of 1-to-1 alignment blocks (bp)	43,744,204 (91.91%)	43,264,255 (96.17%)	43,553,388 (95.21%)	43,595,318 (96.51%)	46,020,467 (101.05%)
Average identity of 1-to-1 alignment blocks (%)	98.44	98.49	98.49	99.01	99.98
Total for all structural variants ≥ 50 bp	777	800	812	568	16
Total number of bases (bp)	2,257,514 (4.74%)	1,680,767 (3.74%)	2,682,990 (5.87%)	1,618,783 (3.58%)	1,528
Total for all structural variants excluding repeat variation	565	611	571	432	15
Total number of bases (bp)	1,160,972 (2.49%)	910,579 (2.02%)	1,269,567 (2.78%)	836,644 (1.85%)	1,476

Table S15: Differentially expressed genes (DEGs) identified in each *Pm. minimum* isolate between rotating and stationary cultures (adj. *P*-value < 0.05).

	**UCR-PA7**	**Pm1118**	**Pm1119**	**Pm449**
**Total DEGs**	5,507	2,777	7,140	7,872
BGC members	2,029	943	2,640	2,907
Inserted genes (%)*	92 (56%)	22 (24%)	0	83 (65%)
Deleted genes (%)**	17 (27%)	8 (7%)	50 (34%)	23 (34%)
**Physically clustered and co-expressed DEG genes (adjacent genes per cluster ≥ 3)**	1,140	466	2,018	2,487
BGC members	473	217	820	932
Inserted genes flanked by at least one Pm1119 gene	8	0	0	12
Inserted genes co-regulated by their own	29	3	0	3
Deleted genes flanked by at least one Pm1119 gene**	4	2	10	9
Deleted genes co-regulated by their own**	3	0	3	3

* For inserted genes, percentages in parenthesis were calculated relative to the total number of inserted genes in each isolate.
** For deleted genes, statistics refer to DE genes that are deleted in other isolates.

Table S16: Number of co-expressed genomic clusters (≥ 3 adjacent genes) in each *Pm. minimum* isolate.

	**UCR-PA7**		**Pm1118**		**Pm1119**		**Pm449**	
**Number of genes/per genomic cluster**	**Over-expressed in rotating condition**	**Under-expressed in rotating condition**	**Over-expressed in rotating condition**	**Under-expressed in rotating condition**	**Over-expressed in rotating condition**	**Under-expressed in rotating condition**	**Over-expressed in rotating condition**	**Under-expressed in rotating condition**
**3**	91	117	50	37	156	175	167	200
**4**	27	40	18	4	60	80	73	104
**5**	11	15	6	0	18	25	15	35
**6**	3	6	2	6	3	14	8	24
**7**	1	6	3	0	3	5	4	10
**8**	0	0	1	0	1	5	0	7
**9**	0	0	0	0	0	1	0	3
**10**	0	0	0	1	0	1	1	2
**11**	0	0	0	0	0	1	0	0
**12**	0	0	0	0	0	0	0	1
**13**	0	0	0	0	0	0	0	1
**14**	0	0	0	0	0	0	0	0
**15**	0	1	0	0	0	1	0	0
**Total clusters**	133	185	80	48	241	308	268	387

Supplementary Figures:

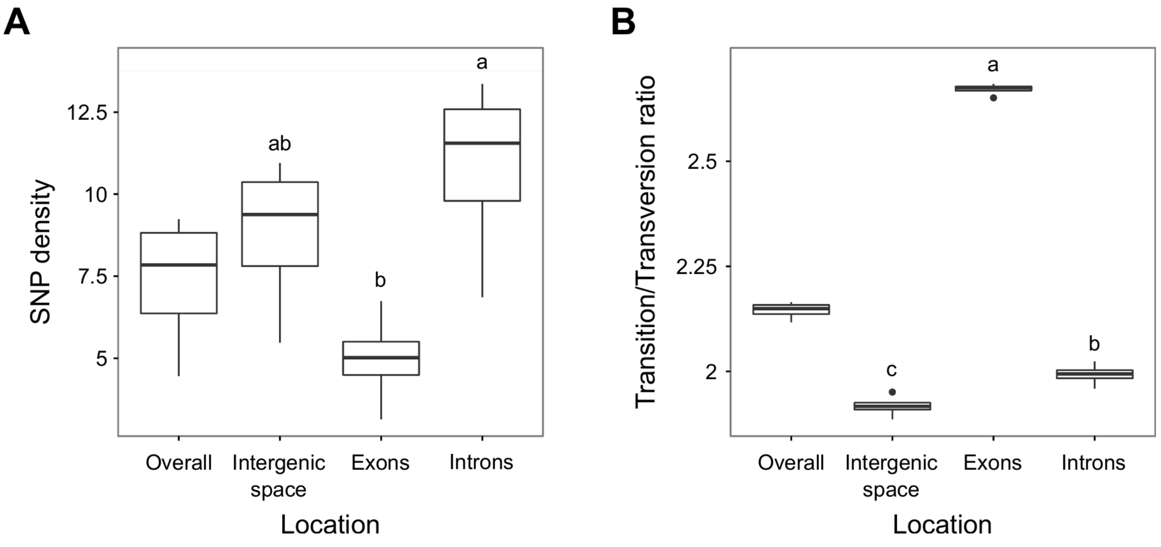

Figure S1: Distribution of Single Nucleotide Polymorphism (SNP) density (**A**) and Transition/Transversion ratio (**B**) in different genomic regions. ANOVA followed by Tukey's post-hoc test was used to compare the SNP density and Transition/Transversion ratio between the intergenic, exonic, and intronic regions. *P*-value < 0.05 was considered statistically significant. Genomic features with the same letter are not significantly different.

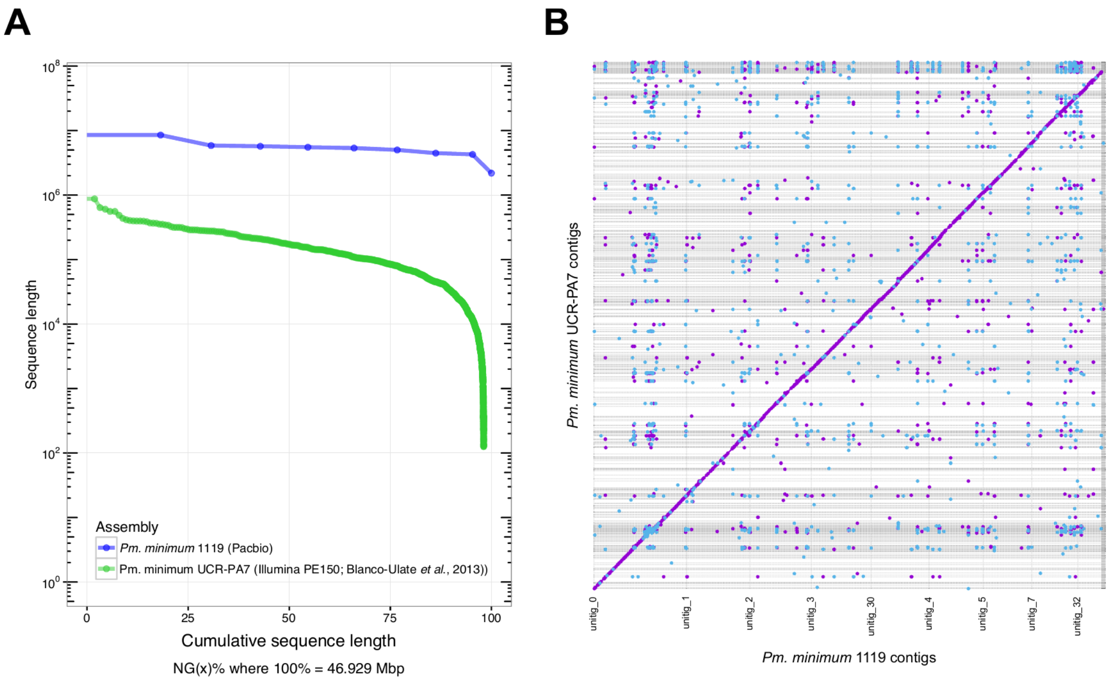

Figure S2: (**A**) Contig length distribution (log10 scale) over the Pm1119 reference genome in the assemblies generated using PacBio reads and Illumina reads (Blanco-Ulate *et al*., 2013). (**B**) Dot plot showing the NUCmer alignments between the contigs of the Pm1119 reference genome and UCR-PA7 contigs (Blanco-Ulate *et al*., 2013).

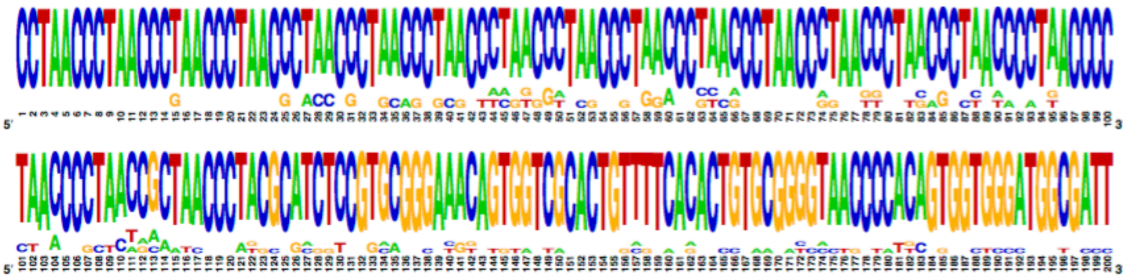

Figure S3: Graphical representation of telomere sequences found at the ends of the 9 longest contigs of the Pm1119 reference genome. Figure was prepared using WebLogo (Crooks *et al.*, 2004).

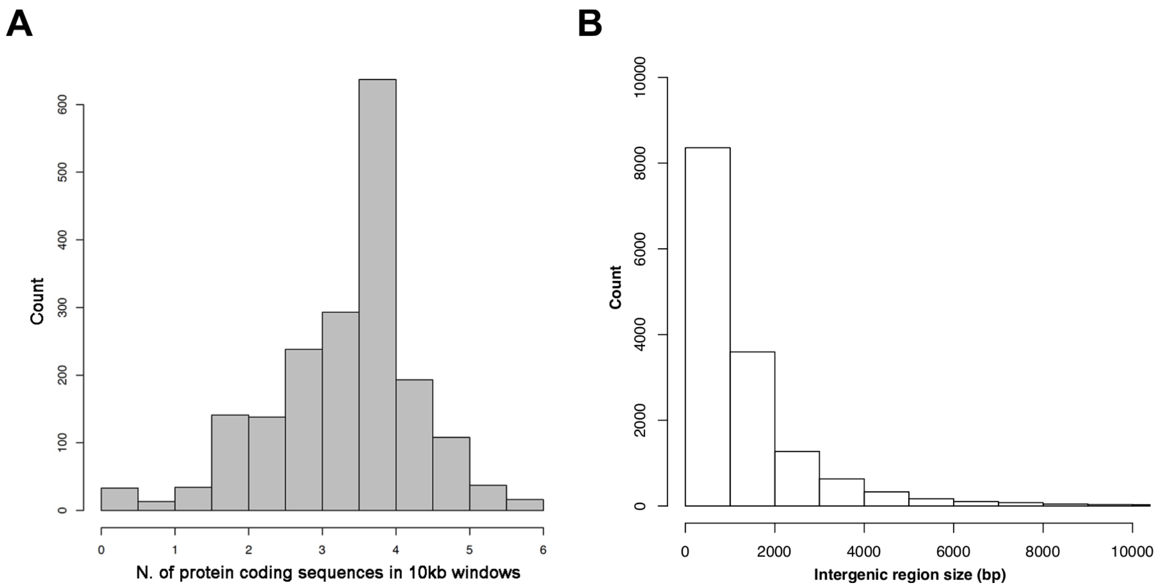

Figure S4: Distribution of the number of protein-coding sequences in 10kb windows (**A**) and of the intergenic region size (**B**) in the Pm1119 reference genome.

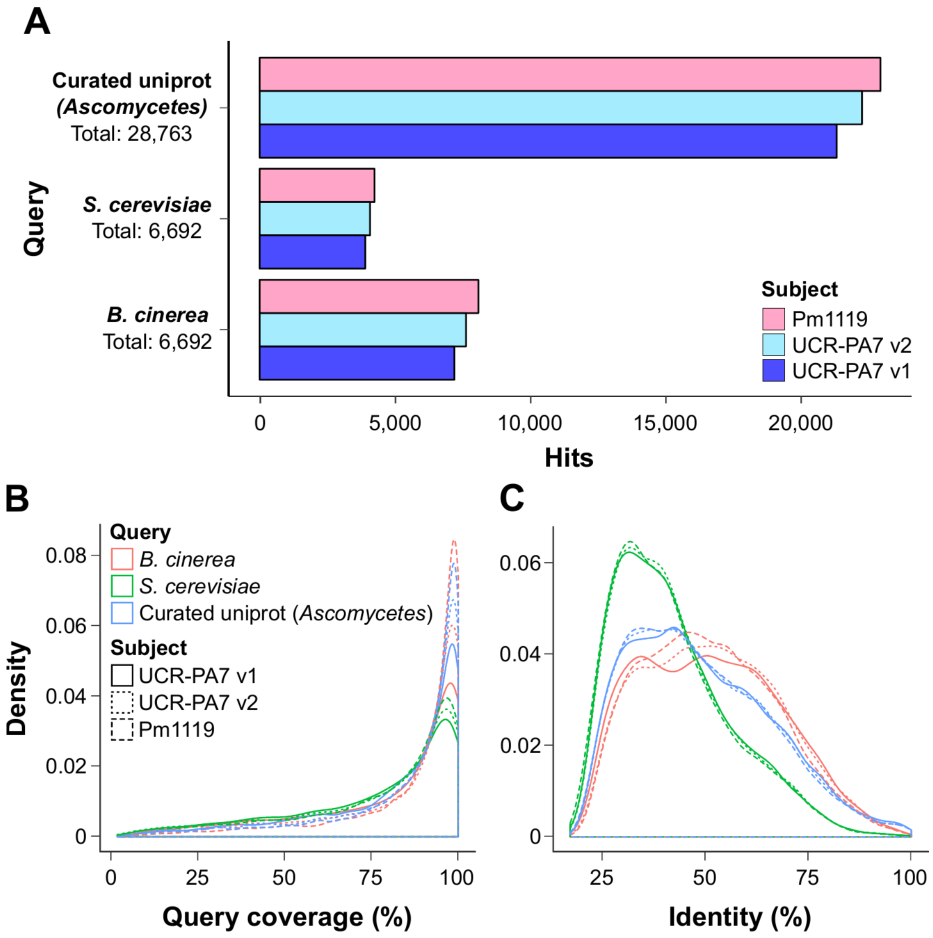

Figure S5: Improvement of the gene prediction in the Pm1119 reference genome compared to previous versions of annotation of UCR-PA7. (**A**) Comparison of the total number of curated UniProt, *Saccharomyces cerevisiae*, and *Botrytis cinerea* proteins that matched the first (*ab initio* only, v1; Blanco-Ulate *et al*., 2013) and the second (v2; Morales-Cruz *et al*., 2015) versions of the UCR-PA7 predicted proteome, and the Pm1119 proteome (BLASTp, e-value < $1e^{-6}$). (**B**) Density distribution of the alignment coverage of curated UniProt, *S. cerevisiae*, and *B. cinerea* proteins matching proteins in the v1 and v2 UCR-PA7 predicted proteomes and Pm1119 predicted proteome (BLASTp, e-value < $1e^{-6}$). (**C**) Density distribution of the similarity between curated uniprot, *S. cerevisiae*, and *B. cinerea* proteins matching proteins in the v1 and v2 UCR-PA7 predicted proteomes and Pm1119 predicted proteome (BLASTp, e-value < $1e^{-6}$).

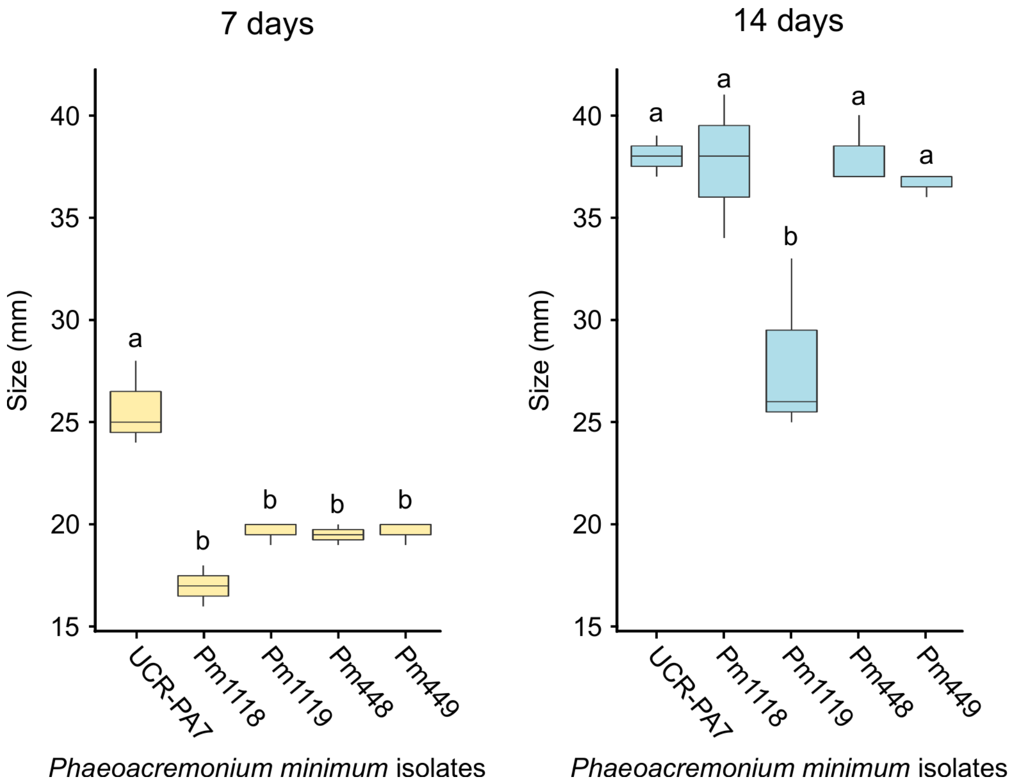

Figure S6: Size of the *Pm. minimum* colonies (mm) after 7 and 14 days of culture on Potato Dextrose Agar medium at 25ºC in the dark. ANOVA followed by Tukey's post-hoc test was used to compare the colony size between the five isolates at each time point. Adjusted *P*-value < 0.05 was considered statistically significant. At each time point, isolates with the same letter are not significantly different in size.

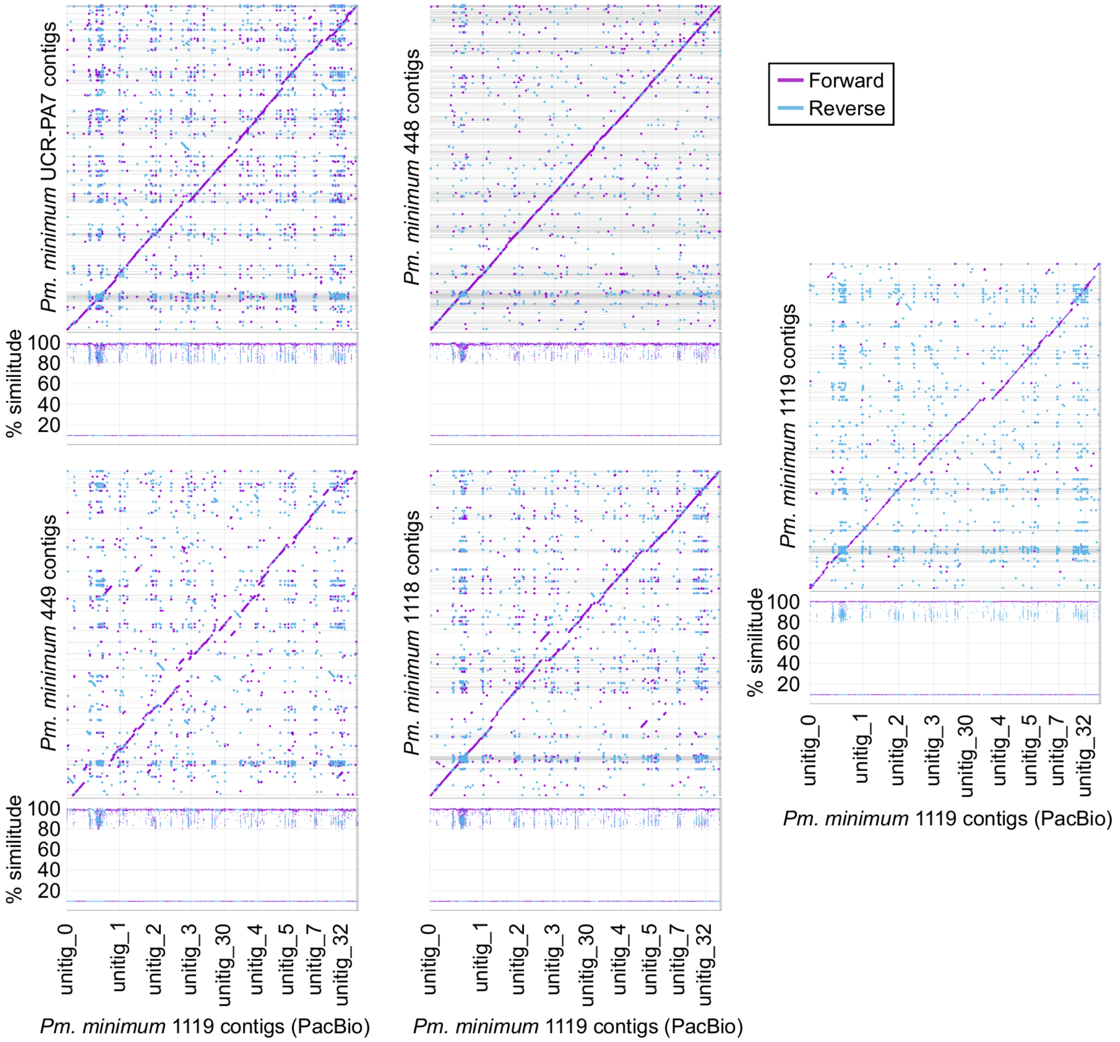

Figure S7: Dotplot view of the NUCmer genome alignments between the *Pm. minimum* 1119 reference genome and the genomes of the other *Pm. minimum* isolates.

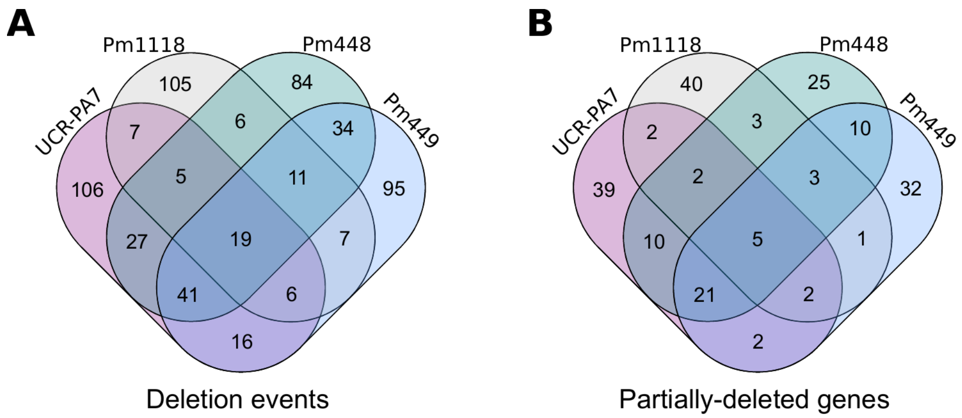

Figure S8: Venn diagrams showing the overlap between deletion events (**A**) and partially-deleted genes (**B**) detected in the four *Pm. minimum* isolates. Overlap between completely deleted genes in show in Figure 4.

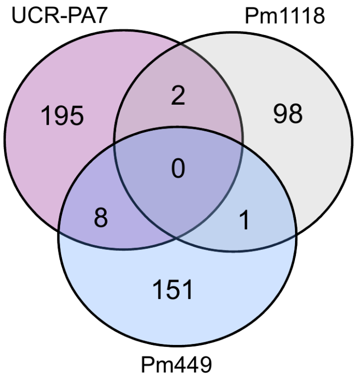

Figure S9: Venn diagram showing the overlap between *de novo* assembled transcripts identified in UCR-PA7, Pm1118, and Pm449, and not found in Pm1119.

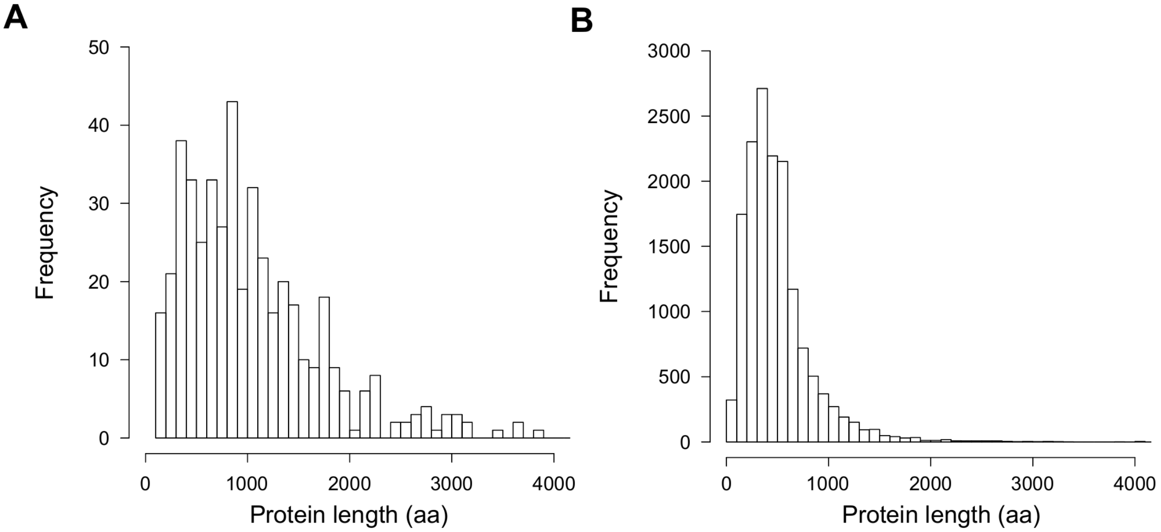

Figure S10: Protein length distribution of the 455 isolate-specific transcripts obtained by *de novo* assembly of UCR-PA7, Pm1118, and Pm449 transcriptomes (**A**), and of the 14,790 predicted proteins of Pm1119 (**B**).

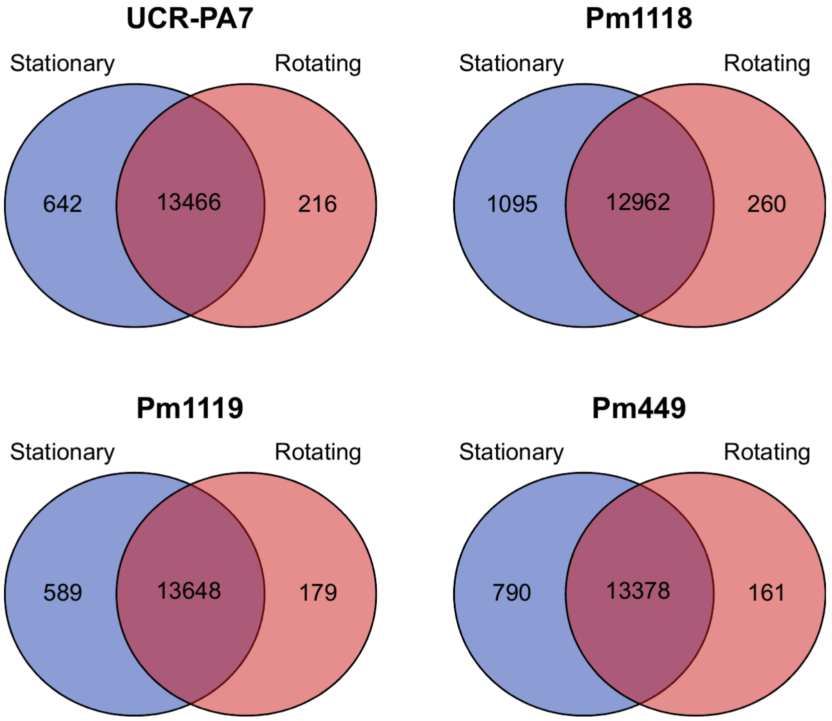

Figure S11: Venn diagrams showing the overlap of the detected protein-coding genes using RNAseq between stationary and rotating conditions.

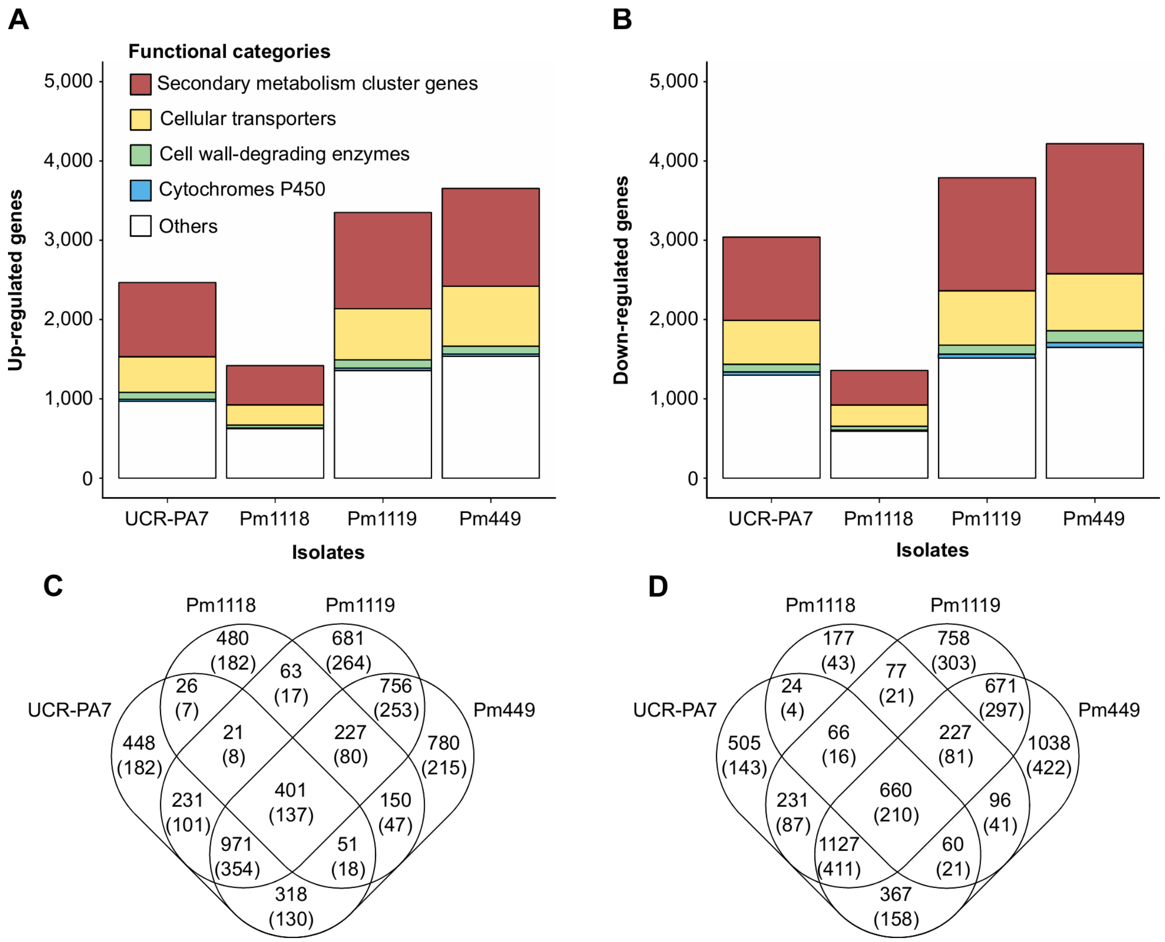

Figure S12: Number of significantly up- (**A**) and down-regulated (**B**) genes (adj. *P*-value < 0.05) when comparing rotating and stationary culture conditions, and their corresponding functional categories. Venn diagrams showing the overlap of significantly up- (**C**) and down-regulated (**D**) genes among isolates. Number of genes belonging to BGCs are represented in parenthesis.

References:

Ademark, P., Varga, A., Medve, J., Harjunpaa, V., Drakenberg, T., Tjerneld, F. *et al.* (1998). Softwood hemicellulose-degrading enzymes from *Aspergillus niger*: purification and properties of a beta-mannanase. *J Biotechnol* 63(3), 199–210.

Ademark, P., de Vries, R.P., Hagglund, P., Stalbrand, H., Visser, J. (2001). Cloning and characterization of *Aspergillus niger* genes encoding an alpha-galactosidase and a beta-mannosidase involved in galactomannan degradation. *Eur J Biochem* 268(10), 2982–2990.

Bankevich, A., Nurk, S., Antipov, D., Gurevich, A.A., Dvorkin, M., Kulikov, A.S., *et al.* (2012). SPAdes: A New Genome Assembly Algorithm and Its Applications to Single-Cell Sequencing. *J Comput Biol* 19(5), 455–477.

Beeson, W.T., Phillips, C.M., Cate, J.H., Marletta, M.A. (2012). Oxidative cleavage of cellulose by fungal copper-dependent polysaccharide monooxygenases. *J Am Chem Soc* 134, 890–892.

Benen, J.A., Kester, H.C., Parenicová, L., Visser, J. (2000). Characterization of *Aspergillus niger* pectate lyase A. *Biochemistry* 39(50), 15563–15569.

Blanco-Ulate, B., Rolshausen, P., Cantu, D. (2013). Draft genome sequence of the Ascomycete *Phaeoacremonium aleophilum* Strain UCR-PA7, a Causal Agent of the Esca Disease Complex in Grapevines. *Genome Announc* 1(3), e00390–13.

Bussink, H.J., Buxton, F.P., Fraaye, B.A., de Graaff, L.H., Visser, J. (1992). The polygalacturonases of *Aspergillus niger* are encoded by a family of diverged genes. *Eur J Biochem* 208(1), 83–90.

Cantarel, B.L., Coutinho, P.M., Rancurel, C., Bernard, T., Lombard, V., Henrissat, B. (2009). The Carbohydrate-Active EnZymes database (CAZy): an expert resource for Glycogenomics. *Nucleic Acids Res* 37, D233–D238.

Crooks, G.E., Hon, G., Chandonia, J.M., Brenner, S.E. (2004). WebLogo: a sequence logo generator. *Genome Res* 14(6), 1188–1190.

Dan, S., Marton, I., Dekel, M., Bravdo, B.A., He, S., Withers, S.G. *et al.* (2000). Cloning, expression, characterization, and nucleophile identification of family 3, *Aspergillus niger* beta-glucosidase. *J Biol Chem* 275(7), 4973–4980.

Flipphi, M.J., Visser, J., van der Veen, P., de Graaff, L.H. (1993a). Cloning of the *Aspergillus niger* gene encoding alpha-l-arabinofuranosidase A. *Appl Microbiol Biotechnol* 39(3), 335–340.

Flipphi, M.J., Panneman, H., van der Veen, P., Visser, J., de Graaff, L.H. (1993b). Molecular cloning, expression and structure of the endo-1,5-alpha-l-arabinase gene of *Aspergillus niger*. *Appl Microbiol Biotechnol* 40(2-3), 318–326.

Gielkens, M.M., Visser, J., de Graaff, L.H. (1997). Arabinoxylan degradation by fungi: characterization of the arabinoxylan-arabinofuranohydrolase encoding genes from *Aspergillus niger* and *Aspergillus tubingensis*. *Curr Genet* 31(1), 22–29.

Gielkens, M.M., Dekkers, E., Visser, J., de Graaff, L.H. (1999). Two cellobiohydrolase-encoding genes from *Aspergillus niger* require d-xylose and the xylanolytic transcriptional activator XlnR for their expression. *Appl Environ Microbiol* 65(10), 4340–4345.

Gysler, C., Harmsen, J.A., Kester, H.C., Visser, J., Heim, J. (1990). Isolation and structure of the pectin lyase D-encoding gene from *Aspergillus niger*. *Gene* 89(1), 101–108.

Harmsen, J.A., Kusters-van Someren, M.A., Visser, J. (1990) Cloning and expression of a second *Aspergillus niger* pectin lyase gene (pelA): indications of a pectin lyase gene family in *A. niger*. *Curr Genet* 18(2), 161–166.

den Herder, I.F., Rosell, A.M., van Zuilen, C.M., Punt, P.J., van den Hondel, C.A. (1992). Cloning and expression of a member of the *Aspergillus niger* gene family encoding alpha-galactosidase. *Mol Gen Genet* 233(3), 404–410.

Kester, H.C., and Visser, J. (1990). Purification and characterization of polygalacturonases produced by the hyphal fungus *Aspergillus niger*. *Biotechnol Appl Biochem* 12(2), 150–160.

Khanh, N.Q., Ruttkowski, E., Leidinger, K., Albrecht, H., Gottschalk, M. (1991). Characterization and expression of a genomic pectin methyl esterase-encoding gene in *Aspergillus niger*. *Gene* 106(1), 71–77.

Krengel, U., and Dijkstra, B.W. (1996). Three-dimensional structure of endo-1,4-beta-xylanase I from *Aspergillus niger*: molecular basis for its low pH optimum. *J Mol Biol* 263(1), 70–78.

Kubicek, C.P., Starr, T.L., Glass, N.L. (2014). Plant cell wall-degrading enzymes and their secretion in plant-pathogenic fungi. *Annu Rev Phytopathol* 52, 427–451.

Kumar, V., Ramakrishnan, S., Teeri, T.T., Knowles, J.K., Hartley, B.S. (1992.) *Saccharomyces cerevisiae* cells secreting an *Aspergillus niger* beta-galactosidase grow on whey permeate. *Biotechnology (N Y)* 10(1), 82–85.

Kurtz, S., Phillippy, A., Delcher, A. L., Smoot, M., Shumway, M., Antonescu, C., *et al.* (2004). Versatile and open software for comparing large genomes. *Genome Biol* 5(2), R12.

Kusters-van Someren, M., Flipphi, M., de Graaff, L., van den Broeck, H., Kester, H., Hinnen, A., *et al*. (1992). Characterization of *the Aspergillus niger* pelB gene: structure and regulation of expression. *Mol Gen Genet* 234(1), 113–120.

Levasseur, A., Asther, M., Record, E. (2005). Overproduction and characterization of xylanase B from *Aspergillus niger*. *Can J Microbiol* 51(2), 177–183.

Martens-Uzunova, E.S., Zandleven, J.S., Benen, J.A., Awad, H., Kools, H.J., Beldman, G., *et al*. (2006). A new group of exo-acting family 28 glycoside hydrolases of *Aspergillus niger* that are involved in pectin degradation. *Biochem J* 400(1), 43–52.

Martens-Uzunova, E.S., Schaap P.J. (2009). Assessment of the pectin degrading enzyme network of *Aspergillus niger* by functional genomics. *Fungal Genet Biol* 46 (Suppl 1), S170–S179.

Massonnet, M., Morales-Cruz, A., Figueroa-Balderas, R., Lawrence, D.P., Baumgartner, K., Cantu, D. (2018). Condition-dependent co-regulation of genomic clusters of virulence factors in the grapevine trunk pathogen *Neofusicoccum parvum*. *Mol Plant Pathol* 19(1), 21–34.

Möller, M., and Stukenbrock, E.H. (2017). Evolution and genome architecture in fungal plant pathogens. *Nat Rev Microbiol* 15(12), 771.

Morales-Cruz, A., Amrine, K.C., Blanco-Ulate, B., Lawrence, D.P., Travadon, R., Rolshausen, P.E., *et al*. (2015). Distinctive expansion of gene families associated with plant cell wall degradation, secondary metabolism, and nutrient uptake in the genomes of grapevine trunk pathogens. *BMC Genomics* 16, 469.

Nattestad, M., and Schatz, M.C. (2016). Assemblytics: a web analytics tool for the detection of assembly-based variants. *Bioinformatics* 32, 3021–3023.

Parenicova, L., Benen, J.A., Kester, H.C., Visser, J. (1998). pgaE encodes a fourth member of the endopolygalacturonase gene family from *Aspergillus niger*. *Eur J Biochem* 251(1-2), 72–80.

Parenicova, L., Benen, J.A., Kester, H.C., Visser, J. (2000a). pgaA and pgaB encode two constitutively expressed endopolygalacturonases of *Aspergillus niger*. *Biochem J* 345(Pt 3), 637–644.

Parenicova, L., Kester, H.C., Benen, J.A., Visser, J. (2000b). Characterization of a novel endopolygalacturonase from *Aspergillus niger* with unique kinetic properties. *FEBS Lett* 467(2-3), 333–336.

Parra, G., Bradnam, K., Ning, Z., Keane, T., Korf, I. (2009). Assessing the gene space in draft genomes. *Nucleic Acids Res* 37(1), 289–297.

Quinlan, R.J., Sweeney, M.D., Lo Leggio, L., Otten, H., Poulsen, J.C., Johansen, K.S., *et al*. (2011). Insights into the oxidative degradation of cellulose by a copper metalloenzyme that exploits biomass components. *Proc Natl Acad Sci USA* 108, 15079–15084.

van Peij, N.N., Brinkmann, J., Vrsanska, M., Visser, J., de Graaff, L.H. (1997). Beta-xylosidase activity, encoded by *xlnD*, is essential for complete hydrolysis of xylan by *Aspergillus niger* but not for induction of the xylanolytic enzyme spectrum. *Eur J Biochem* 245(1), 164–173.

van Peij, N.N., Gielkens, M.M., de Vries, R.P., Visser, J., de Graaff, L.H. (1998). The transcriptional activator XlnR regulates both xylanolytic and endoglucanase gene expression in *Aspergillus niger*. *Appl. Environ. Microbiol.* 64(10), 3615–3619.

Pel, H.J., de Winde, J.H., Archer, D.B., Dyer, P.S., Hofmann, G., Schaap, P.J., *et al*. (2007). Genome sequencing and analysis of the versatile cell factory *Aspergillus niger* CBS 513.88. *Nat Biotechnol* 25(2), 221–231.

Raffaele, S., and Kamoun, S. (2012). Genome evolution in filamentous plant pathogens: why bigger can be better. *Nat Rev Microbiol* 10(6), 417–430.

Simão, F.A., Waterhouse, R.M., Ioannidis, P., Kriventseva, E.V., Zdobnov, E.M. (2015). BUSCO: Assessing genome assembly and annotation completeness with single-copy orthologs. *Bioinformatics* 31(19), 3210–3212.

Suykerbuyk, M.E., Kester, H.C., Schaap, P.J., Stam, H., Musters, W., Visser, J. (1997). Cloning and characterization of two rhamnogalacturonan hydrolase genes from *Aspergillus niger*. *Appl Environ Microbiol* 63(7), 2507–2515.

van der Vlugt-Bergmans, C.J., Meeuwsen, P.J., Voragen, A.G., van Ooyen, A.J. (2000). Endo-xylogalacturonan hydrolase, a novel pectinolytic enzyme. *Appl Environ Microbiol* 66(1), 36–41.

de Vries, R.P., Michelsen, B., Poulsen, C.H., Kroon, P.A., van den Heuvel, R.H., Faulds, C.B., *et al*. (1997). The faeA genes from *Aspergillus niger* and *Aspergillus tubingensis*

encode ferulic acid esterases involved in degradation of complex cell wall polysaccharides. *Appl Environ Microbiol* 63(12), 4638–4644.

de Vries, R.P., van den Broeck, H.C., Dekkers, E., Manzanares, P., de Graaff, L.H., Visser, J. (1999). Differential expression of three alpha-galactosidase genes and a single beta-galactosidase gene from *Aspergillus niger*. *Appl Environ Microbiol* 65(6), 2453–2460.

de Vries, R.P., Kester, H.C., Poulsen, C.H., Benen, J.A., Visser, J. (2000). Synergy between enzymes from Aspergillus involved in the degradation of plant cell wall polysaccharides. *Carbohydr Res* 327(4), 401–410.

de Vries, R.P., Jansen, J., Aguilar, G., Parenicova, L., Joosten, V., Wulfert, F., *et al.* (2002a). Expression profiling of pectinolytic genes from *Aspergillus niger*. *FEBS Lett* 530(1-3), 41–47.

de Vries, R.P., Parenicova, L., Hinz, S.W., Kester, H.C., Beldman, G., Benen, J.A., *et al.* (2002b). The beta-1,4-endogalactanase A gene from *Aspergillus niger* is specifically induced on arabinose and galacturonic acid and plays an important role in the degradation of pectic hairy regions. *Eur J Biochem* 269(20), 4985–4993.

de Vries, R.P., vanKuyk, P.A., Kester, H.C., Visser, J. (2002c). The *Aspergillus niger* faeB gene encodes a second feruloyl esterase involved in pectin and xylan degradation and is specifically induced in the presence of aromatic compounds. *Biochem J* 363(Pt 2), 377–386.
